# ETV4 interacts with LOXL2 to induce epigenetic activation of NID1 during colorectal cancer progression

**DOI:** 10.7150/ijbs.116383

**Published:** 2025-10-20

**Authors:** Tinghui Jiang, Xin Liu, Hao Liu, Kailong Du, Yitao Wang, Hui Fan, Ying Zhang, Lin Cui, Hewei Zhang, Chundong Zhang, Yong Zhu, Zhongyu Liu, Youquan Bu, Yunlong Lei

**Affiliations:** 1 Department of Biochemistry and Molecular Biology, and Molecular Medicine and Cancer Research Center, College of Basic Medical Sciences, Chongqing Medical University, Chongqing 400016, China; 2 Tianfu Jincheng Laboratory, Chengdu, 610093, China; 3 Animal Diseases and Public Health Engineering Research Center of Henan Province, No.6, Tech Avenue, Yibin District, Luoyan, Henan Province, China.; 4 Department of Cell Biology and Genetics, Chongqing Medical University, Chongqing, China.; 5 The Research Department of 989 Hospital. No.2 Huaxia Road, Jianxi distric, Luoyang, Henan Province, China. Luoyang Polytechnic, No.6, Tech Avenue, Yibin District, Luoyang, Henan Province, China.

**Keywords:** colorectal cancer, ETV4, LOXL2, NID1, demethylation

## Abstract

Colorectal cancer (CRC) is one of the most common malignant cancers worldwide and its poor prognosis is mainly caused by metastasis. Although extensive studies, the potential molecular mechanisms of CRC metastasis are not fully understood. In the present study, we found that ETV4 was remarkably upregulated in CRC and its overexpression correlated with lymph node metastasis. ETV4 could significantly promote the growth, epithelial-mesenchymal transition (EMT) and metastasis of CRC cells *in vitro* and *in vivo*. Mechanistic investigations found that LOXL2 was a novel transcriptional target and a direct interacting partner of ETV4 and was vital to ETV4-induced CRC malignant phenotypes. Further studies revealed that ETV4/LOXL2 complex could bind NID1 promoter to mediate its demethylation, induce NID1 expression and subsequent ERK signaling pathway activation, which is required for ETV4/LOXL2-mediated EMT and metastasis of CRC. Meanwhile, the expression of ETV4 and LOXL2 were significantly negatively correlated with the methylation of NID1 promoter in clinical samples. Besides, the combined ETV4, LOXL2 and NID1 as prognostic markers is more reliable than any one alone. Taken together, in this study, we demonstrated that ETV4 played a critical role in CRC metastasis, and unraveled the novel regulatory axis of ETV4/LOXL2/NID1, which contributed to the malignant progression of CRC.

## 1. Introduction

Colorectal cancer (CRC) is one of the most commonly diagnosed cancers among women and men worldwide, and its mortality ranks third among all cancer types^1^-^3^. Recent advancement in oncology has resulted in a better understanding of CRC progression, and early diagnosis combined with clinical therapy has partly reduced the morbidity and mortality of CRC^4^, ^5^. However, the overall outcome of patients with CRC is unsatisfied largely due to metastasis, including lymphatic and distant metastasis^6^. Therefore, there is an urgent demand to identify new CRC biomarkers to further reveal the underlying metastatic mechanism of CRC.

E26 transformation-specific (ETS) family is a classic and evolutionarily conserved transcription factor family^7^, which regulates the expression of a variety of genes in embryonic development, including branching morphogenesis of lung and kidney, neuronal pathfinding and embryonic limb bud^8^, ^9^. ETS variant 4 (ETV4) is an important member of ETS transcription factor family, and constitutes the PEA3 subfamily together with ETV1, ETV5. ETV4 was found medium-high expression in prostate cancer, breast cancer, non-small cell lung cancer and digestive tract cancer, including oral cancer, esophageal cancer, gastric and colorectal cancer, and was negatively correlated with patient prognosis and survival^10^-^13^. It is reported that ETV4 can promote tumor invasion and metastasis by upregulation of matrix metalloproteinase (MMP). In addition, ETV4 has also been found to induce the transcription of uPA, COX2 and SCCA to promote the invasion and metastasis of tumor cells^13^. Besides regulating transcription, ETV4 also promotes the metastasis in non-small cell lung cancer by activating the Rho/ROCK signaling pathway^11^ and prostate cancer by activating the PI3K and RAS signaling pathways^12^. However, the mechanism of how ETV4 mediates colorectal cancer cell proliferation and metastasis still needs to be further revealed.

Lysyl oxidase-like 2 (LOXL2) is an important member of the lysyl oxidase (LOX) family, a type of secreted copper- and quinone-dependent amine oxidases, which mainly catalyzes the cross-linking of collagen and elastin in the extracellular matrix and plays an important role in the biosynthesis of the connective tissue matrix^14^-^16^. However, subsequent studies found that the members of the lysyl oxidase family, including LOXL2, also distribute in the nucleus and play an essential role in transcription regulation^15^-^17^. The role of LOXL2 in tumorigenesis and development is complex and tissue-specific. LOXL2 was first found to be downregulated in fibroblasts, head and neck squamous cell carcinoma, lung adenocarcinoma and serous ovarian carcinoma in RAS-transformed mice, and was once considered as a tumor suppressor gene^15^, ^16^. However, the following studies have found that LOXL2 was highly expressed in breast cancer, colorectal cancer, esophageal cancer, bile duct cancer, liver cancer, pancreatic cancer and gastric cancer, and was closely associated with metastasis and prognosis^16^-^19^. Moreover, LOXL2 can promote the invasion and metastasis of cancer cells by inducing EMT in colorectal cancer, breast cancer and liver cancer^16^-^19^. The mechanism of LOXL2 regulation in the cell invasion, metastasis and EMT in different cancers is still not very clear. Some studies have suggested that it promoted gastric cancer cell metastasis mainly by interacting with other proteins such as MARCKSL1 and thereby activating FAK/SRC and integrin signaling pathways^17^, or binding to the transcription factor SNAIL in the nucleus inhibited E-cadherin expression and promoted EMT in breast cancer^18^, ^20^. In addition, LOXL2 can inhibit E-cadherin expression by binding and oxidizing H3K4me3 in breast cancer cells^15^.

NID1, a member of the extracellular matrix binding protein nidogen family (also known as Entactin), is a secretory glycoprotein and an important component of the basal membrane (BM)^21^. The nidogens family is one of the main components of basement membrane. As a specialized extracellular matrix thin layer, BM regulates almost all cellular events like adhesion, migration, differentiation, proliferation and apoptosis^22^. Nidogens are able to bind laminin-1 and collagen type IV to form a ternary complex, which serves in establishing and maintaining the structure of basal membrane. Nidogens also bind integrins on cell membranes to establish cell adhesion. Loss of nidogens loosens cell interaction with basal membranes and favors the invasion and metastasis of cancer cells^21^-^25^. Zhou *et al.* reported that abnormal expression of NID1 changes the overall structure and function of the basal membrane and could mediate the metastasis and chemoresistance in ovarian cancer cells by activating FAK/ERK signaling pathway^21^. Our previous studies also showed that NID1 was highly expressed in the serum of ovarian cancer patients and could be a potential blood marker for this malignant disease^21^, ^26^.

In the present study, we confirmed that the expression of ETV4 is indeed upregulated in CRC, which is closely related to lymph node metastasis and cancer stage. ETV4 overexpression could transactivate LOXL2 and recruit it to the promoter region of NID1 to mediate the demethylation and expression of NID1, which in turn activates ERK pathway and promotes CRC cell growth, metastasis and EMT. In this study, we demonstrate that ETV4 plays a vital role in CRC progression, and that this novel axis of ETV4/LOXL2/NID1 may be valuable for early diagnosis and treatment of CRC patients.

## 2. Material and Methods

### 2.1 Data acquisition and bioinformatics

Transcriptome data, methylation data and clinical data were downloaded from The Cancer Genome Atlas (TCGA) data portal (https://cancergenome.nih.gov/) and GENE EXPRESSION OMNIBUS (GEO) database. For the reliability of the subsequent analysis, the transcriptome gene expression matrix was filtered according to the following criteria: Eliminate over 90% of the genes with 0 expression level in the sample. Genes without corresponding annotation information were then removed. Differential genes were obtained by the differential analysis in R DEseq2 package and limma package. The significance of gene expression difference was identified by using absolute fold change (FC) >1.8. Protein-protein interaction network (PPI) was constructed using STRING database version 11.5. Molecular Complex Detection (MCODE) is a plugin for Cytoscape 3.6.1 that discovers the dense regions that interact in the PPI network. From the modules identified by the MCODE score, the module with the highest MCODE score was determined. Gene Set Enrichment Analysis (GSEA) was performed to identify the oncogenic signatures and significantly changed pathways by GSEA v4.0.3 software. COAD patients were divided into high-risk and low-risk groups and the Kaplan-Meier survival curve was generated that predicted cases with low or high risk by GEPIA (Gene Expression Profiling Interactive Analysis) database.

### 2.2 Cell culture

Human CRC cell lines, HCT116 (colon cancer cell, ras mutation), RKO (colorectal cancer cell, wild-type p53), and HT29 (colorectal cancer, p53 mutation), were purchased from Chinese Academy of Sciences Shanghai cell bank (Shanghai, China). Cells were maintained in a humidified atmosphere containing 5% CO_2_ at 37°C in DMEM (HCT116, HT29) or MEM (RKO) supplemented with 100 units/mL of penicillin, 100 mg/mL of streptomycin and 10% (vol/vol) heat-inactivated FBS (Invitrogen). The last time of authentication for these cells was December 2021.

### 2.3 Tissue microarrays and Immunohistochemistry

For CRC tissue microarray analysis (TMA), we purchased CRC tissue microarray slide from AurageneBioscience Corporation (TC0167, Hunan China) which contained normal colorectal tissues (n=10) and malignant colorectal cancer tissues (n=110). The detailed clinical information of tissue microarray was provided in Supplementary Table S1. Immunohistochemical analysis was conducted as described previously^27^, and the information of the antibodies used was provided in Supplementary Table S2. Informed consent for tissue procurement was obtained from all patients or their families before beginning study, and Ethics approval was gotten from the Institutional Ethics Committee of Chongqing Medical University.

### 2.4 RT-qPCR and Immunoblotting analysis

Total RNA extraction and RT-qPCR were performed as previously described^27^ with the SYBR® Premix Ex Taq TM (TAKARA) and CFX96 thermocycler (Bio-Rad). The cDNA array for CRC (HCRT101) was purchased from Origene Technologies (Rockville, MD, USA), and the clinical pathological information was provided in Supplementary Table S3. For immunoblotting analysis, protein from human colorectal cancer cells were gained with RIPA buffer (Beyotime) supplied with protease inhibitor cocktail (Bimake), then all lysates were analyzed by SDS-PAGE. The information of primers and antibodies used were listed in Supplementary Table S4 and Supplementary Table S2.

### 2.5 Stable ETV4 overexpression and knockdown cell generation

The ETV4 lentiviral overexpression vector as well as empty vector was purchased from Guangzhou GeneCopoeia (Guangzhou, China), and oligonucleotide sequences for ETV4 shRNA were annealed and cloned into the lentiviral shRNA expression vector psi-LVRH1GP (Guangzhou, GeneCopoeia). The lentivirus particles were respectively co-transfected into 293Ta cells to produce recombinant lentiviral particles. After 48h, viral supernatants were collected and infected the target cells. Forty-eight hours after lentivirus infection, cells were selected with puromycin (1μg/mL) for about 72h to generate the stable ETV4 overexpression and knockdown cells^27^, ^28^.

### 2.6 Cell proliferation, migration and invasion assays

Cell proliferation was measured using Cell Counting Kit-8 (CCK8) (Bimake, USA)^27^, ^28^. Wound healing, transwell migration and invasion assays were conducted as described previously^27^, ^28^.

### 2.7 RNA-sequencing analysis

For RNA-seq analysis, the exponentially grown ETV4 stable overexpression as well as its corresponding control cells were collected, total RNA was extracted, and cDNA libraries were then constructed and sequenced as described previously^27^. Differentially expressed genes were subjected to Gene Ontology (GO) Enrichment Analysis and Kyoto Encyclopedia of Genes and Genomes (KEGG) Pathway analyses. Gene set enrichment analysis (GSEA; http://www.broad.mit.edu/gsea) was conducted to identify the significantly changed pathways and oncogenic signatures.

### 2.8 Indirect immunofluorescence (IF)

The indirect immunofluorescence assays were performed as described previously^29^. Cells were fixed and incubated with primary antibodies (Supplemental Table S2) followed by the incubation with fluorescently-conjugated secondary antibodies. The nucleus was stained with medium containing 4'6-diamidino-2-phenylindole (DAPI, Sigma).

### 2.9 Tumor xenografts

Cells were subcutaneously injected into the dorsal flank of four- to six-week-old nude mice (BALB/c). All nude mice were randomly divided into different groups (n=4). Tumor size and tumor weight were measured and calculated as described previously, and the tumor volume was calculated using the equation L × W^2^/ 2 (L = length, W = width)^27^, ^28^, ^30^. All animals were humanely treated in accordance with the guidelines of the Institutional Animal Care and Treatment Committee of Chongqing medical University.

### 2.10 siRNA synthesis and transfection

The negative control siRNA and siRNAs targeting ETV4, LOXL2 and NID1 were chemically synthesized by Shanghai GenePhama (Shanghai, China). The sequences of siRNAs used were listed in Supplementary Table S5. Then siRNAs were transfected into CRC cells by using Lipofectamine RNAiMAX reagent (Invitrogen) according to the manufacturer's instruction.

### 2.11 Luciferase reporter constructs and site-directed mutagenesis

The LOXL2 promoter region (-1651/+122) and the NID1 promoter (-2111/+244) were obtained by PCR-based amplification and cloned into pGL3-Basic vector to generate reporters, LOXL2-P1773 and NID1-P2355, respectively. A series of luciferase reporter mutants harboring mutations in ETV4 binding sites were constructed by the site-directed mutagenesis kit (TOYOBO, Osaka, Japan) based on the two parental constructs of LOXL2-P1773 and NID1-P2355 as described previously^31^-^33^. The sequences of primers used were listed in Supplementary Table S6. All the constructs were validated by DNA sequencing by Sangon Biotech (Shanghai, China). Cells were seeded in triplicate into 12-well plates and co-transfected with the corresponding plasmids and pGL4.74[hRluc/TK] (Promega), then luciferase activities were measured using Dual-Luciferase Assay System (Promega) as described previously^27^, ^31^, ^33^, ^34^.

### 2.12 Protein-protein docking analysis

The 3D structures of ETV4 and LOXL2 were downloaded from RCSB Protein Data Bank online tool (http://www.rcsb.org/pdb/home/home.do). Protein-protein docking analysis was carried out using the HDOCK server (http://hdock.phys.hust.edu.cn/), which provides the three top models of possible interactions between two proteins^35^, ^36^.

### 2.13 Proximity ligation assay (PLA)

The PLA assay was conducted with the Duolink *in situ* PLA Kit (Sigma-Aldrich, Munich, Germany) according to the manufacturer's instructions. PLA signals, recognized as red fluorescent dots, were visualized and images were captured by the confocal microscopy (Leica TCS SP8)^27^.

### 2.14 Co-immunoprecipitation (co-IP) assay

For co-immunoprecipitation analysis, the Flag-ETV4 expression plasmids and HA-LOXL2 expression plasmids were transiently transfected into 293Ta cells. After 48h, cells were lysed with IP lysis buffer (Beyotime). The Protein A/G Magnetic Beads (Bimake) were pre-incubated with primary anti-Flag antibody (Sigma) and normal mouse IgG (Millipore) for 1h at 4℃, then incubated with the cell extracts overnight at 4℃. The immunoprecipitated protein complexes were washed three times with cold PBST. The bound proteins were eluted by boiling in loading buffer before analyzed with Western blotting. The truncated constructs of Flag-ETV4-P1(1-339), Flag-ETV4-P2(278-484) and Flag-ETV4-P3(340-484) were generated by PCR using Flag-ETV4 as the template. The sequences of primers used were listed in Supplementary Table S6.

### 2.15 Glutathione-S-transferase pull-down assay

The pGEX-GST-ETV4 vector was purchased from GeneCreate (Wuhan, China), and the truncated constructs of GST-ETV4-P1 was generated by PCR using GST-ETV4 as the template. The sequences of primers used were listed in Supplementary Table S6. Glutathione-S-transferase (GST), GST-ETV4 or GST-ETV4-P1 fusion proteins were induced with 0.5 mM isopropylthiogalactoside (IPTG) (Shengon Biotech, Shanghai, China) for 20 hours at 20℃, then purified according to manufacturer's instructions (Beyotime, China). For GST pull-down assay, LOXL2 proteins was expressed in HCT116-ETV4 cells and protein were harvested with RIPA buffer (Beyotime, China) supplied with protease inhibitor cocktail (Bimake). Then the LOXL2 protein was incubated with GST, GST-ETV4 or GST-ETV4-P1 bound to glutathione-Sepharose beads (Beyotime, China), and the adsorbed proteins were analyzed by immunoblotting analysis^37^.

### 2.16 Chromatin immunoprecipitation (ChIP)

ChIP was performed with EZ-Magna Chromatin immunoprecipitation A/G Kit (Millipore) according to manufacturer's instructions. The sequences of primers were supplied in Supplemental Table S4.

### 2.17 DNA isolation, bisulfite conversion, MSP and Pyrosequencing

The whole DNA was extracted using Tissue DNA Kit (OMEGA) according to the manufacturer's instruction. And then these DNA samples were proceeded with bisulfite modification and purified using the EpiTect Fast Bisulfite Conversion Kits (QIAGEN). The methylation status of NID1 promoter was detected by Methylation Specific PCR (MSP) and RT-qPCR. The sequences of primers were supplied in Supplemental Table S4. Meanwhile, the bisulfite-converted DNA was pyrosequenced to quantify the methylated sites of the NID1 promoter^22^.

### 2.18 Statistical analysis

All data were statistically analyzed using the SPSS 16.0 software (SPSS INC, Chicago, IL, USA). Qualitative variables were compared using Chi-square tests whereas quantitative variables were analyzed by Student's t-test. The distribution of data was expressed as mean ± SD. The difference between different groups was evaluated by Student's t tests or Mann-Whitney U tests. Data were analyzed using the GraphPad Prism software version 8.30 (GraphPad software, San Diego California USA) and *P* <0.05 was considered statistically significant.

## 3. Results

### 3.1 ETV4 is upregulated in human colorectal cancers and associated with progress

**Figure 1:**
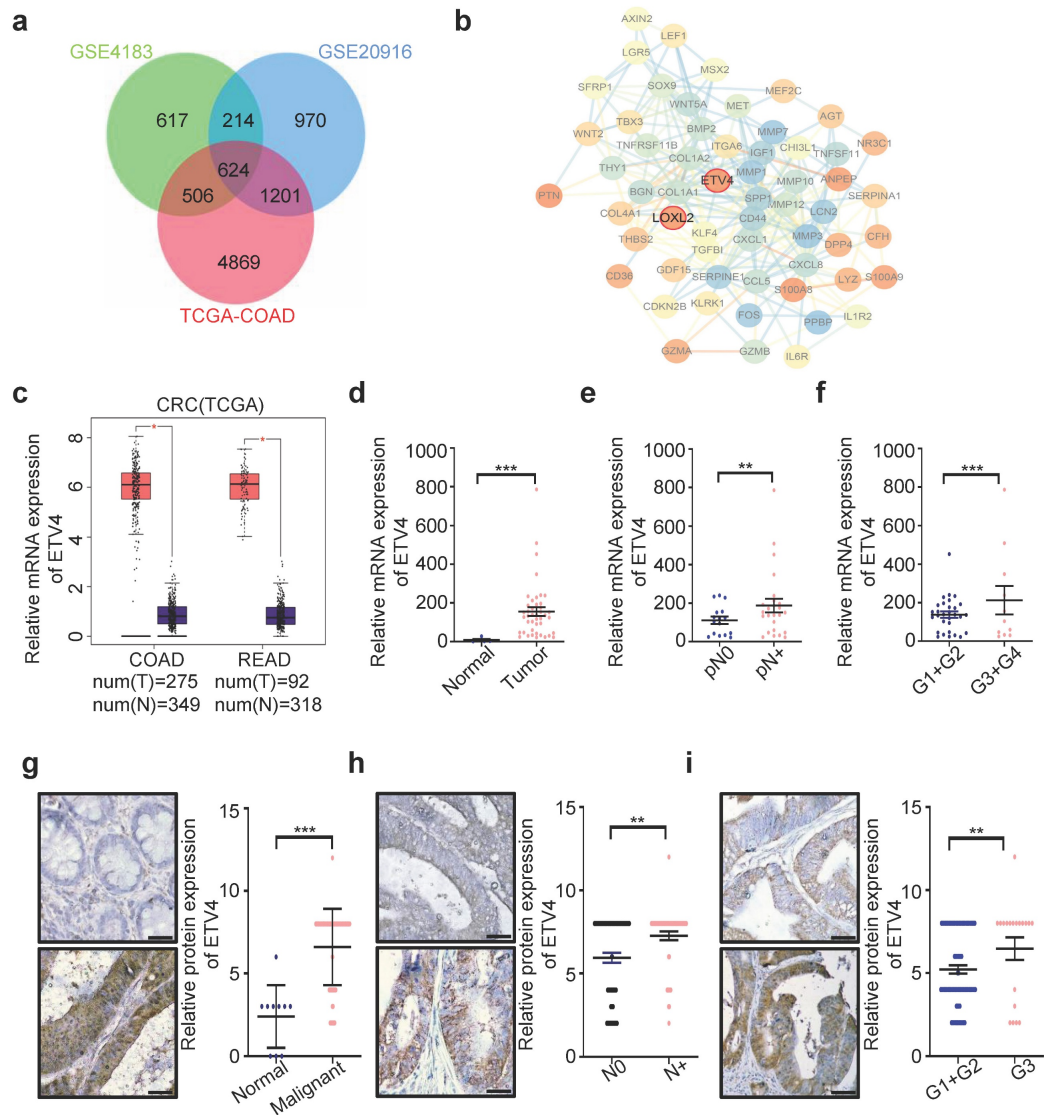
** Upregulation of ETV4 is related with progression of CRC. a.** Venn diagram of differential expression genes (DEGs) overlapped among the three datasets (GSE4183, GSE20916 and TCGA-COAD). **b.** The complete PPI network of common DEGs in CRC. Each circle represents one gene, and each line indicates one protein-protein interaction. Figure inside the circle indicates the corresponding protein structure. **c.** ETV4 mRNA overexpression in CRC. The TCGA ETV4 expression data were analyzed online by GEPIA. (COAD, Colon adenocarcinoma; READ, Rectum adenocarcinoma). **d.** Expression of ETV4 mRNA was detected via RT-qPCR with specific primers in CRC cDNA array purchased from OriGene. **e-f.** The correlation of ETV4 mRNA expression with lymphatic metastasis (p<0.001) or pathologic grade (p<0.001) was analyzed by RT-qPCR using CRC cDNA array. **g.** ETV4 protein expression was verified by immunohistochemical (IHC) assay using CRC tissue microarray slides (p<0.001), scale bar: 25μm.** h-i.** The association of ETV4 protein expression with lymphatic metastasis (p<0.01) or pathologic grade (p<0.01) was analyzed by immunohistochemistry and quantitative immunohistochemistry analysis, scale bar: 25μm. *p < 0.05, **p < 0.01, *** p < 0.001.

To identify the new CRC biomarkers for diagnosis and therapy, we utilized three datasets (GSE4183, GSE20916 and TCGA-COAD) to screen for differential expression genes (DEGs) (Fig.S1a-1d), and obtained the DEGs overlapped in three datasets (Fig.1a and Supplementary file 1). Surprisingly, among these genes, lots of genes were defined as transcription factors (TFs) (Supplementary Table S7). In consideration of the importance of transcription factors in cancer therapy, thus we focused on TFs for further analysis. Among all potential targets, ETV4, a member of the ETS transcription factor family, caught our attention, because ETV4 expression showed the greater variation than other TFs (Supplementary Table S7). Next, the interaction network analysis was employed to explore whether there are some regulatory relationships among those TFs and DEGs, and results showed that ETV4 was associated with so many DEGs and located at the core position of the interaction network (Fig.1b), which indicated that ETV4 may play an important role in CRC progression. Moreover, quantitative analysis by Gene Expression Profiling Interactive Analysis (GEPIA, http://gepia.cancer-pku.cn/) confirmed that ETV4 mRNA expression levels were significantly upregulated in CRC relative to normal colorectal tissues (Fig.1c). To confirm the above bioinformatics results, the human CRC cDNA array from Origene was used to detect the ETV4 mRNA expression, and the results showed that ETV4 was indeed remarkably elevated in CRC samples compared with the normal colorectal tissues (p<0.001, Fig.1d), and of note, the expression level of ETV4 mRNA was positively correlated with lymphatic metastasis (p<0.001, Fig.1e) and pathologic grades (p<0.001, Fig.1f). Furthermore, the protein expression summary analysis for ETV4 via the Human Protein Atlas (HPA https://www.proteinatlas.org/) database revealed that most cancer tissues displayed moderate to strong nuclear staining, in a few cases accompanied by weak cytoplasmic immunoreactivity (Fig.S2). Consistent with cDNA array results, IHC analysis on a colon cancer tissue microarray revealed that the expression of ETV4 protein was upregulated in CRC tissues compared to normal tissues (p<0.001, Fig.1g and Supplementary Table S8) and the expression of ETV4 protein was significantly associated with lymphatic metastasis (p<0.01, Fig.1h and Supplementary Table S8) and pathologic grades (p<0.01, Fig.1i and Supplementary Table S8). Taken together, these analyses indicated that the expression of ETV4 is associated with CRC metastasis and pathologic grades, suggesting ETV4 may play a role in CRC progression.

### 3.2 ETV4 promotes CRC cell growth and metastasis *in vitro* and *in vivo*

**Figure 2:**
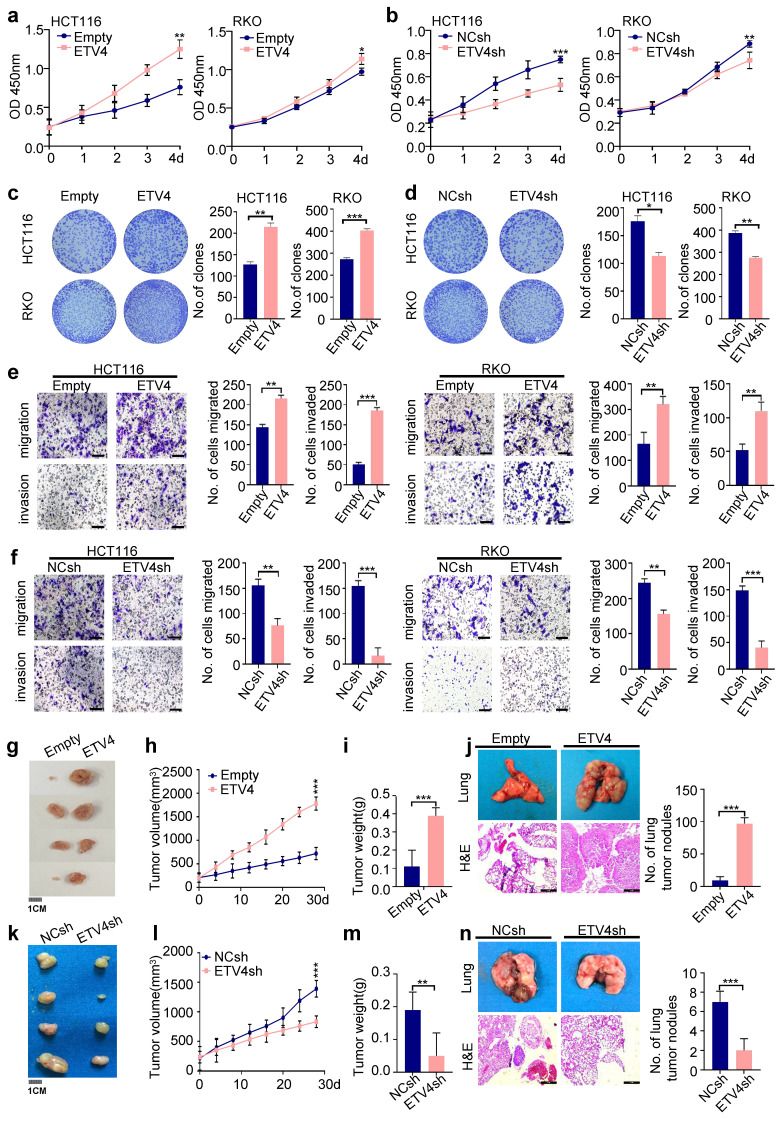
** ETV4 enhances colorectal cancer cell proliferation and metastasis *in vitro* and *in vivo*. a-b.** Cell viability was determined by CCK8 assay in stable ETV4 overexpression or knockdown HCT116 and RKO cells in indicated time points. **c-d.** Cell proliferation was analyzed by colony formation assay. HCT116 and RKO cells were stable overexpression or knockdown ETV4, and cells were seeded into 6-well plates for two weeks and colony numbers were quantified. Representative images for colony formation of indicated cells (left). Quantification of clone numbers (right). **e-f.** Transwell migration and invasion assay of HCT116 and RKO with stable overexpression or knockdown ETV4. Representative images for transwell migration and invasion assays of indicated cells (left), scale bar: 100μm. Relative number of migrated or invaded cells (right). **g-i, k-m.** HCT116 cells with stable ETV4 overexpression or knockdown were injected subcutaneously into nude mice (n=4). The images of isolated tumors derived from mice (g, k). The tumor size was measured 1-2 times a week for tumor growth curve construction (h, l) and the weight of tumors were measured at time of sacrificed (i, m). **j, n.** Stable ETV4 overexpression or knockdown HCT116 cells were injected into nude mice through the tail vein. The H&E staining shows the lung metastases in mice and quantification of number of metastases, scale bar: 25μm. Data represent the mean ± SD. All experiments were performed in triplicates. *p < 0.05, **p < 0.01, *** p < 0.001.

To investigate the role of ETV4 in CRC progression, we constructed HCT116, RKO, and HT29 cells stably expressing ETV4, as well as stable knockdown of ETV4 (Supplementary Fig.S3a-b). Overexpression of ETV4 led to notably increased the cell viability in these CRC cell lines, conversely, knockdown of ETV4 resulted in opposite effects (Fig.2a-b and Supplementary Fig.S3c). Consistently, ETV4 could markedly enhance the proliferation of colorectal cancer cells, as evidenced by colony formation assays (Fig.2c-d and Supplementary Fig.S3d). Furthermore, the results of wound healing assay, transwell migration and invasion showed that ETV4 overexpression exerted a promoting effect on CRC cell migration and invasion while knockdown of ETV4 attenuated cell migration and invasion (Fig.2e-f and Fig.S3e-h). Collectively, these results indicated that ETV4 promotes colorectal cancer cells growth and invasion *in vitro*.

The impact of ETV4 on tumor growth and distant metastasis was then investigated *in vivo*. A mouse xenograft model was conducted by inoculating subcutaneously HCT116 cells into nude mice. Consistent with *in vitro* study, ETV4 overexpression significantly promoted CRC growth, as evidenced by larger size of tumors and faster growth rate compared with the control group (Fig.2g-2i). In contrast, ETV4 knockdown reduced HCT116 cell-derived xenograft tumor growth (Fig.2k-2m). In addition, we analyzed the level of PCNA in tumor samples by immunoblot and immunohistochemical staining, which affirmed that ETV4 promoted colorectal cancer proliferation *in vivo* (Fig.S4a-d). Moreover, HCT116 cells were injected into nude mice through the tail vein to develop a nude mouse metastatic model. As expected, overexpression of ETV4 in the HCT116 cells significantly promoted metastasis in lung tissues whereas knockdown of ETV4 impaired metastasis *in vivo* (Fig.2j, 2n). Together, these data suggested that ETV4 could accelerate colorectal tumor growth and metastasis *in vivo*.

### 3.3 ETV4 induces EMT in CRC cells

**Figure 3:**
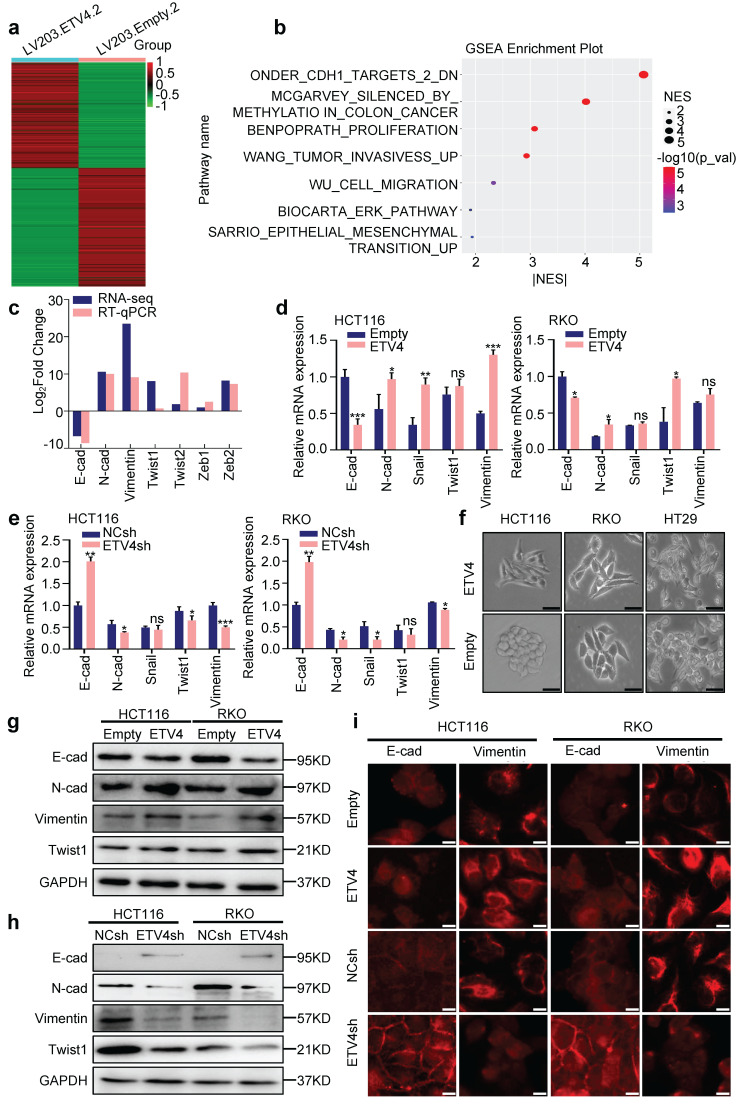
** ETV4 mediates EMT in colorectal cancer cells. a.** RNA-seq analysis for stable ETV4 overexpression HCT116 cells. Heatmap shows the differentially expressed genes. **b.** Gene set enrichment analysis (GSEA) based on the RNA-seq data. **c.** Verification of EMT markers based on the RNA-seq data. **d-e.** Verification of the expression changes of E-cadherin, N-cadherin, Snail, Twist1 and Vimentin using RT-qPCR in HCT116 and RKO cells with stable ETV4 overexpression (d) or knockdown (e). **f.** Images of the cell morphology of HCT116, RKO and HT29 cells with stable ETV4 overexpression, scale bar: 50μm.** g-h.** Immunoblot analysis of the expression levels of E-cadherin, N-cadherin, Twist1 and Vimentin in HCT116 and RKO cells with stable ETV4 overexpression (g) or knockdown (h). **i.** Confirmation of E-cadherin and Vimentin through immunofluorescence assay in HCT116 and RKO cells with stable ETV4 overexpression or knockdown, scale bar: 50μm.

To further reveal the potential mechanisms underlying ETV4-induced CRC metastasis, RNA sequencing (RNA-seq) analysis was performed to comprehensively examine the differential expression genes in stable ETV4 overexpression and control cells (Fig.3a), 6814 genes were up-regulated and 8036 genes were down-regulated in HCT116-ETV4 cells (|log2FC| ≥ 2.0) (Supplementary file 2). Next, the GSEA-4.0.3V software was used for gene set enrichment analysis (GSEA), and a number of gene sets were enriched, including tumor invasion (WANG_TUMOR_INVASIVESS_UP), cell migration (WU_CELL_MIGRATION) and epithelial-mesenchymal transition (EMT) (SARRIO_EPITHELIAL_MESENCHYMAL_TRANSITION_UP) (Fig.3b), which was consistent with the previous results that ETV4 promotes colorectal cancer cells metastasis (Fig.2). In addition, the gene set ONDER_CDH1_TARGETS_2_DN (Fig.S5a) was enriched indicating that ETV4 may be associated with EMT. Given in the importance of EMT in tumor metastasis, we first explored the association of ETV4 expression and CRC EMT. The RNA-seq and RT-qPCR analysis indicated that ETV4 overexpression suppressed the epithelial marker (E-cadherin) and upregulated the mesenchymal markers (N-cadherin, Vimentin and Twist1) (Fig.3c-3d and Fig.S5b). Conversely, knockdown of ETV4 led to the opposite effects (Fig.3e and Fig.S5c). Consistently, ETV4 overexpression converted the epithelial phenotype to a mesenchymal-like phenotype in colorectal cancer cells (Fig.3f). Moreover, Western blotting was conducted to detect EMT changes after ETV4 overexpression or knockdown. In accordance with the RT-qPCR results, overexpression of ETV4 downregulated the expression of E-cadherin and enhanced the expression of mesenchymal markers, such as N-cadherin, Vimentin and Twist1 (Fig.3g and Fig.S5d). In contrast, silencing ETV4 resulted in the reverse effects (Fig.3h and Fig.S5d). Immunofluorescence analysis further determined that the observed EMT changes in colorectal cancer cell lines (Fig.3i and Fig.S5e). Taken together, these results confirmed that ETV4 could induce EMT in colorectal cancer cell lines.

### 3.4 LOXL2 is a novel transcriptional target gene of ETV4 in CRC cells

**Figure 4:**
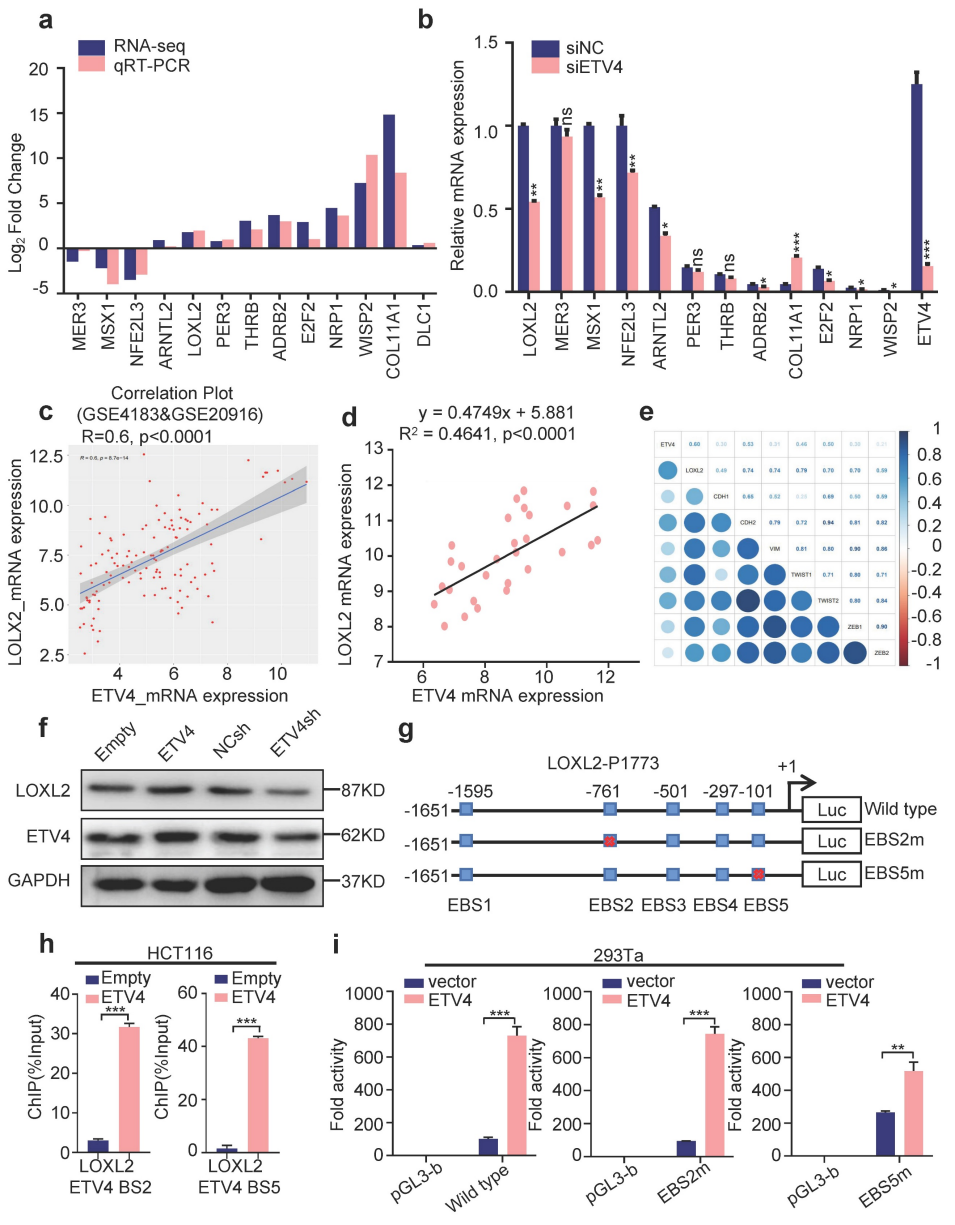
** LOXL2 is a new target gene of ETV4. a.** Verification of ETV4-regulated downstream genes which were involved in EMT in stable ETV4 overexpression HCT116 cells. **b.** Determination of ETV4-regulated downstream genes which were involved in EMT by RT-qPCR in ETV4 knockdown HCT116 cells. **c.** Confirmation of correlation between ETV4 mRNA and LOXL2 mRNA via GSE4183 and GSE20916. **d.** Verification of the relation of ETV4 and LOXL2 by RT-qPCR using the human CRC cDNA array. **e.** Determination of association between EMT markers and ETV4 or LOXL2 via GSE4183 and GSE20916. The size and color intensity of the circles encode the correlation magnitude: larger circles with darker hues indicate stronger correlations, while smaller, lighter circles represent weaker associations. **f.** Immunoblotting was conducted to determine ETV4 and LOXL2 expression in stable ETV4 overexpression and knockdown HCT116 cells. **g.** Schematic illustration of the wildtype and mutant LOXL2-P1773 luciferase (Luc) promoter reporters. The transcription site for LOXL2 gene is indicated as +1. The ETV4 binding sites are shown as boxes. The mutated sites are crossed. **h.** ChIP assay. Chromatin fragments were prepared from HCT116 cells and immunoprecipitated with anti-ETV4 antibody or control IgG. The precipitated DNA was then amplified by real-time PCR with primers directed to the ETV4 binding sites in the LOXL2 promoter region. **i.** Luciferase reporter assays. 293Ta cells were transiently transfected with the indicated plasmids, and forty-eight hours after transfection, the luciferase activities were measured.

To identify the molecular mechanism underlying the ETV4-mediated EMT and metastasis in colorectal cancer cells, we next leveraged our RNA-seq data and a series of key downstream genes associated with EMT were further confirmed, including MER3, MSX1, LOXL2, THRB, WISP2, COL11A1, ADRB2 and E2F2 (Fig.4a-b). Among the downstream genes, LOXL2, is of particular interest for the further study, considering it was proved to be a biomarker in CRC (Fig.1a, Fig.S6a-b), as well as it was in the same cluster of protein-protein interaction (PPI) network as ETV4 (Fig.1b). Thus, the correlation between ETV4 and LOXL2 was explored, and results showed that LOXL2 and ETV4 were highly correlated in GSE4183 and GSE20916 datasets (Fig.4c), as well as the human CRC cDNA array (Fig.4d). Besides, the combined Kaplan-Meier analysis showed that patients with both upregulation of ETV4 and LOXL2 were remarkably associated with poorer disease-free survival (p=0.023), as compared to those with high ETV4 (p=0.2) or LOXL2 (p=0.16) (Fig.S6c-e). Furthermore, correlation analysis via GSE4183 and GSE20916 datasets showed that LOXL2 was highly correlated with EMT markers, such as CDH1, CDH2, VIMENTIN, TWIST1, TWIST2, ZEB1 and ZEB2 (Fig.4e). Altogether, these data preliminarily suggested that LOXL2 might play an important role in ETV4 mediated EMT and metastasis in CRC.

To verify that LOXL2 is a candidate target gene of ETV4 in CRC, we first analyzed the changes in its protein levels after ETV4 overexpression or knockdown in CRC cell lines. Consistent with the above mRNA expression results, Western blot analysis showed that ETV4 overexpression significantly increased LOXL2 protein level and ETV4 knockdown decreased LOXL2 protein level (Fig.4f). Given ETV4 is a transcription factor and could regulate the expression of many genes. We next analyzed the transcription factor binding sites for human LOXL2 promoter via the Jasper database (http://jaspar.genereg.net/) and the result showed that there are five consensus binding sites for ETV4 in LOXL2 promoter (Fig.4g and Fig. S6f). Then, chromatin immunoprecipitation (ChIP) assay demonstrated that ETV4 could be recruited to LOXL2 promoter region through ETV4-bing site 2 (EBS2) and ETV4-bing site 5 (EBS5) (Fig.4h and Fig. S6g). Next, the LOXL2 promoter region (-1651/+122) was cloned into the firefly luciferase reporter vector, and were co-transfected with pcDNA3.0-ETV4 expression vector into cells. The luciferase reporter assay showed that ETV4 notably increased the LOXL2-P1773 luciferase activity compared with the control plasmid (Fig.4i). Then, we introduced point mutations at EBS2 and EBS5 within the LOXL2-P1773 (-1651/+122) region, respectively (Fig.4g). And luciferase reporter assay determined that mutations of the ETV4 binding sites attenuated the ETV4-mediated transactivation of LOXL2 promoter in EBS5m (Fig.4i), which demonstrated that the EBS5 is required for ETV4 to regulate LOXL2. Collectively, these data verified that ETV4 is directly recruited to the LOXL2 promoter via ETV4 binding sites and activates the transcription of LOXL2.

### 3.5 LOXL2 is required for ETV4-induced malignant phenotypes in CRC cells

To investigate whether LOXL2 contributes to the malignant phenotypes in CRC, we first interrogated the role of LOXL2 in CRC cells through *in vitro* study. Silencing LOXL2 led to decreased the cell viability in CRC cells, as evidenced by CCK8 assay (Fig.S7a-c). Furthermore, transwell migration and invasion assay (Fig.S7d), and wound healing assay (Fig.S7e) were performed to indicate that LOXL2 knockdown attenuated cell migration and invasion. In addition, Western blotting demonstrated that knockdown of LOXL2 upregulated the expression of E-cadherin and decreased the expression of mesenchymal markers, such as Vimentin and Twist1 (Fig.S7f). Collectively, our data determined that LOXL2 indeed plays a role in promoting colorectal cancer cells growth and invasion.

**Figure 5:**
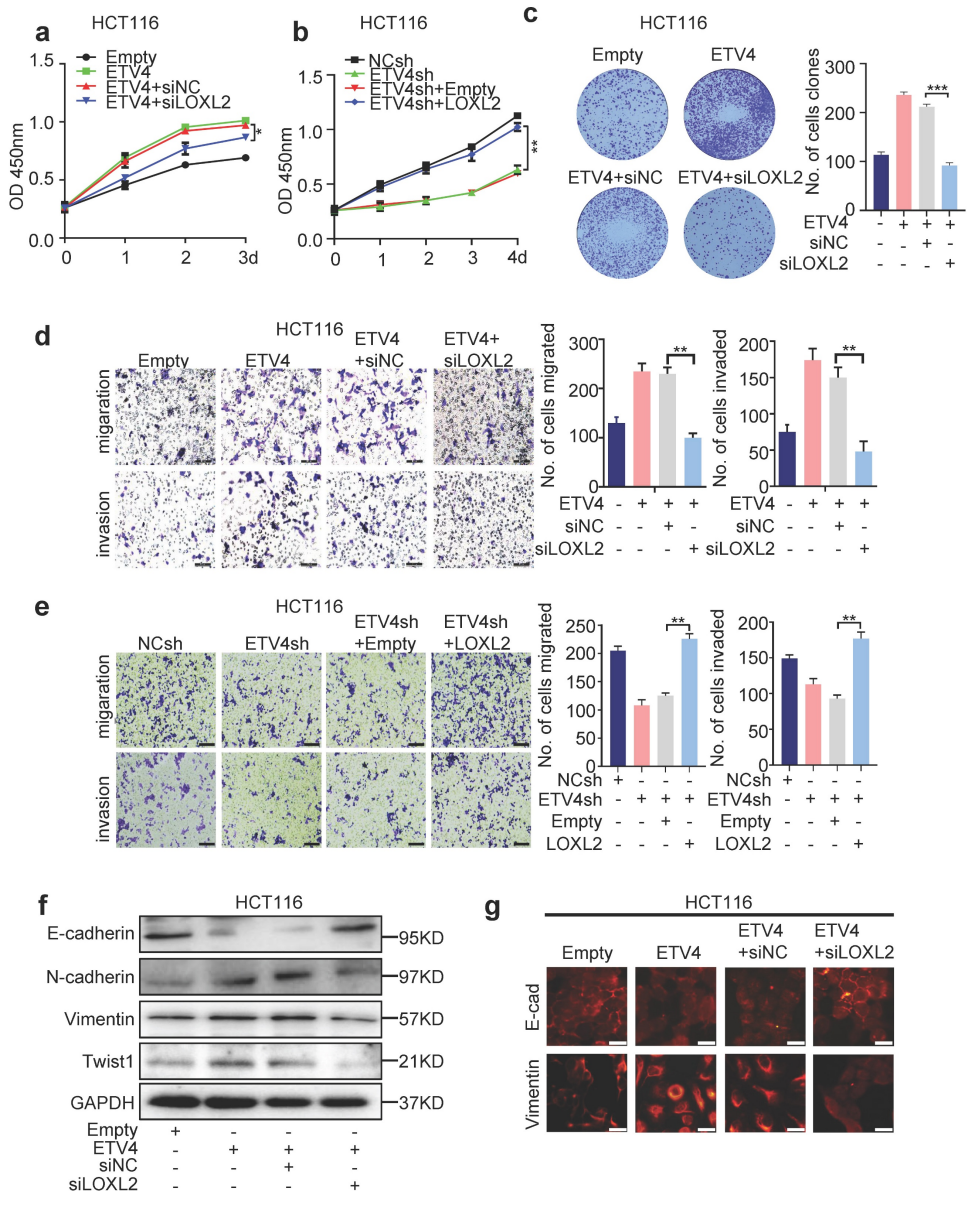
** LOXL2 is essential for ETV4-mediated malignant phenotypes in CRC cells. a.** Stable ETV4 overexpression HCT116 cells were transiently transfected with negative siRNA or LOXL2 siRNA, and cell viability was determined by CCK8 kit. **b.** Stable ETV4 knockdown HCT116 cells were transiently transfected with pcDNA3.1 empty or pcDNA3.1-HA-LOXL2 expression constructs. The cell viability was determined by CCK8 assay at the indicated time points. **c, d.** HCT116 cells were treated as described in (a), and cells were subjected to colony formation assay (c), transwell migration and invasion assay (d). **e.** HCT116 cells were treated as described in (b), then cells were subjected to transwell migration and invasion assay (e, scale bar: 100μm). **f.** Immunoblot analysis of E-cadherin, N-cadherin, Twist1 and Vimentin expression in HCT116 cells treated as described in (a) for 24h. **g.** Immunofluorescence analysis of E-cadherin and Vimentin expression in HCT116 cells treated as described in (a), scale bar: 25μm. Data represent the mean ± SD. All experiments were performed in triplicates. *p < 0.05, **p < 0.01.

Next, to explore the essential role of LOXL2 in ETV4-mediated CRC malignant phenotypes, we first downregulated LOXL2 expression in stable overexpression ETV4 CRC cell lines (Fig.S8a-b). The CCK8 assay showed that silencing LOXL2 could abrogate the proliferation enhanced by ETV4 overexpression, while overexpression of LOXL2 rescued the cell viability inhibited by ETV4 knockdown in CRC cells (Fig 5a-b and Fig.S9a-b). Consistently, LOXL2 knockdown could markedly inhibit the proliferation of ETV4-overexpression colorectal cancer cells, as evidenced by colony formation assays (Fig.5c and Fig.S9c). Moreover, we conducted transwell migration and invasion assay, and wound healing assay in CRC cells. The results showed that LOXL2 knockdown impaired the ETV4-induced enhancement of cell migration and invasion (Fig.5d and Fig.S9d, 9f, 9h), while LOXL2 overexpression restored the ETV4 knockdown-mediated inhibition of migration affects (Fig.5e and Fig.S9e, 9g, 9i) in CRC cells. In addition, Western blotting was performed to detect EMT changes after LOXL2 knockdown in stable overexpression ETV4 cells. And the results indicated that silencing of LOXL2 exerted an inhibited effect on ETV4-promoted EMT progress, by suppressing the expression of mesenchymal markers, such as N-cadherin, Vimentin and Twist1, and enhancing the expression of E-cadherin (Fig.5f and Fig.S9j). Immunofluorescence analysis further confirmed the observed EMT changes in HCT116 cells (Fig.5g). Altogether, these data determined that LOXL2 is essential for ETV4-mediated malignant phenotypes in CRC cells.

### 3.6 NID1 is a downstream gene of ETV4/LOXL2-induced aggressive phenotype in CRC

**Figure 6:**
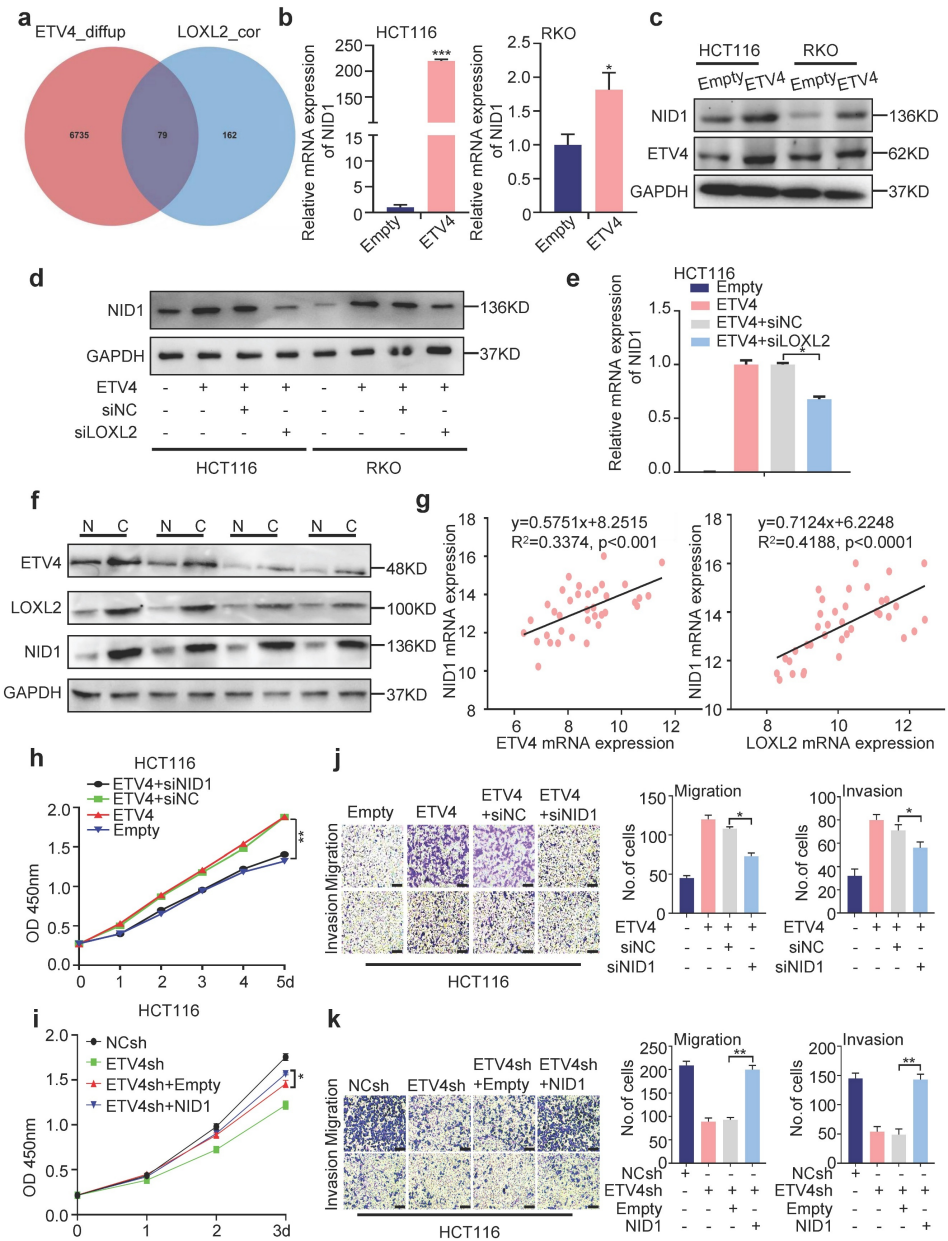
** NIDI is a downstream gene of ETV4/LOXL2-induced aggressive phenotype in CRC. a.** Venn diagram showing the candidate genes of ETV4 and LOXL2. The genes of which the Pearson correlation coefficient with LOXL2 was greater than 0.7, and combined with the differential expression genes (|log2FC| ≥ 2.0) in our previous ETV4 overexpression RNA-seq data, finally yielding 79 candidate genes. **b-c**. Expression of NID1 was determined by RT-qPCR (b) and immunoblot analysis (c) in HCT116 and RKO cells with stable ETV4 overexpression. **d-e.** Expression of NID1 was determined by immunoblot analysis (d) and RT-qPCR (e) when silencing LOXL2 in HCT116 and RKO cells with stable ETV4 overexpression. **f.** Expression of ETV4, LOXL2 and NID1 in paired non-tumor and tumor tissues (n=4) was determined by immunoblot analysis. N, normal tissue; C, cancer tissue. **g.** The correlation of ETV4 or LOXL2 with NID1 was detected via RT-qPCR in CRC cDNA array purchased from OriGene. **h.** Stable ETV4 overexpression HCT116 cells were transiently transfected with negative siRNA or NID1 siRNA. The cell viability was determined by CCK8 assay at the indicated time points. **i.** Stable ETV4 knockdown HCT116 cells were transiently transfected with empty or pcDNA3.0-NID1. The cell viability was determined by CCK8 assay at the indicated time points. **j-k.** HCT116 cells were treated as described in (h) or (i) for 24h, then cells were subjected to transwell migration and invasion assay (i, scale bar: 100μm). Data represent the mean ± SD. All experiments were performed in triplicates. *p < 0.05, **p < 0.01.

To further elucidate how ETV4/LOXL2 promote EMT and metastasis in CRC cells, we first identified 241 genes of which the Pearson correlation coefficient with LOXL2 was greater than 0.7 via TCGA database, and combined with 6814 up-regulated differential expression genes (|log2FC| ≥ 2.0) in our previous ETV4 overexpression RNA-seq data, finally yielding 79 candidate genes (Fig.6a and Supplementary file 3). Next, we chose NID1 for further study because of its highest variation. The RT-qPCR and Western blotting assays determined overexpression of ETV4 promoted NID1 expression (Fig.6b-c), while silencing LOXL2 could attenuate NID1 up-regulation by ETV4 at mRNA and protein levels (Fig.6d-e). Overexpression of LOXL2 alone can also promote the expression of NID1 (Fig.S10a-b).

Furthermore, Western blotting assay indicated that the expression of NID1 was consistent with ETV4 and LOXL2 in paired non-tumor and tumor tissues samples (n=4) (Fig.6f), and the correlation analysis on human CRC cDNA array showed that NID1 was positively correlated with ETV4 and LOXL2, respectively (Fig.6g). Moreover, expression clustering & correlation for LOXL2 via the Human Protein Atlas database also revealed that NID1 was one of the 15 nearest neighbors with LOXL2 based on tissue protein expression (Fig.S11). Besides, the NID1 mRNA expression analysis via three datasets (TCGA-COAD, GSE4183 and GSE20916) showed that NID1 may serve as a biomarker in CRC (Fig.S12a). Importantly, in the combined Kaplan-Meier analysis, patients with simultaneously high expressed ETV4, LOXL2 and NID1 were markedly associated with poorer disease-free survival (p=0.037), as compared to those with high NID1 (p=0.48), ETV4 and NID1 (p=0.69), or LOXL2 and NID1 (p=0.28) (Fig.S12b-e). Moreover, correlation analysis via GSE4183 and GSE20916 datasets showed that NID1 was correlated with EMT markers, such as CDH2, VIMENTIN, TWIST1, TWIST2, ZEB1 and ZEB2 (Fig.S12f). Collectively, the above data demonstrated that NID1 may be a potential key downstream effector of ETV4/LOXL2 in CRC.

To investigate whether NID1 contributes to the malignant phenotypes in CRC, we first explored the role of NID1 in CRC cells through *in vitro* study. NID1 knockdown could inhibit the cell viability in CRC cells, as evidenced by CCK8 assay and colony formation assay (Fig.S13a-d). Furthermore, transwell migration and invasion assay (Fig.S13e), and wound healing assay (Fig.S13f) determined that NID1 knockdown attenuated cell migration and invasion. Taken together, those data determined that NID1 indeed plays a role in promoting colorectal cancer cells growth and invasion.

**Figure 7:**
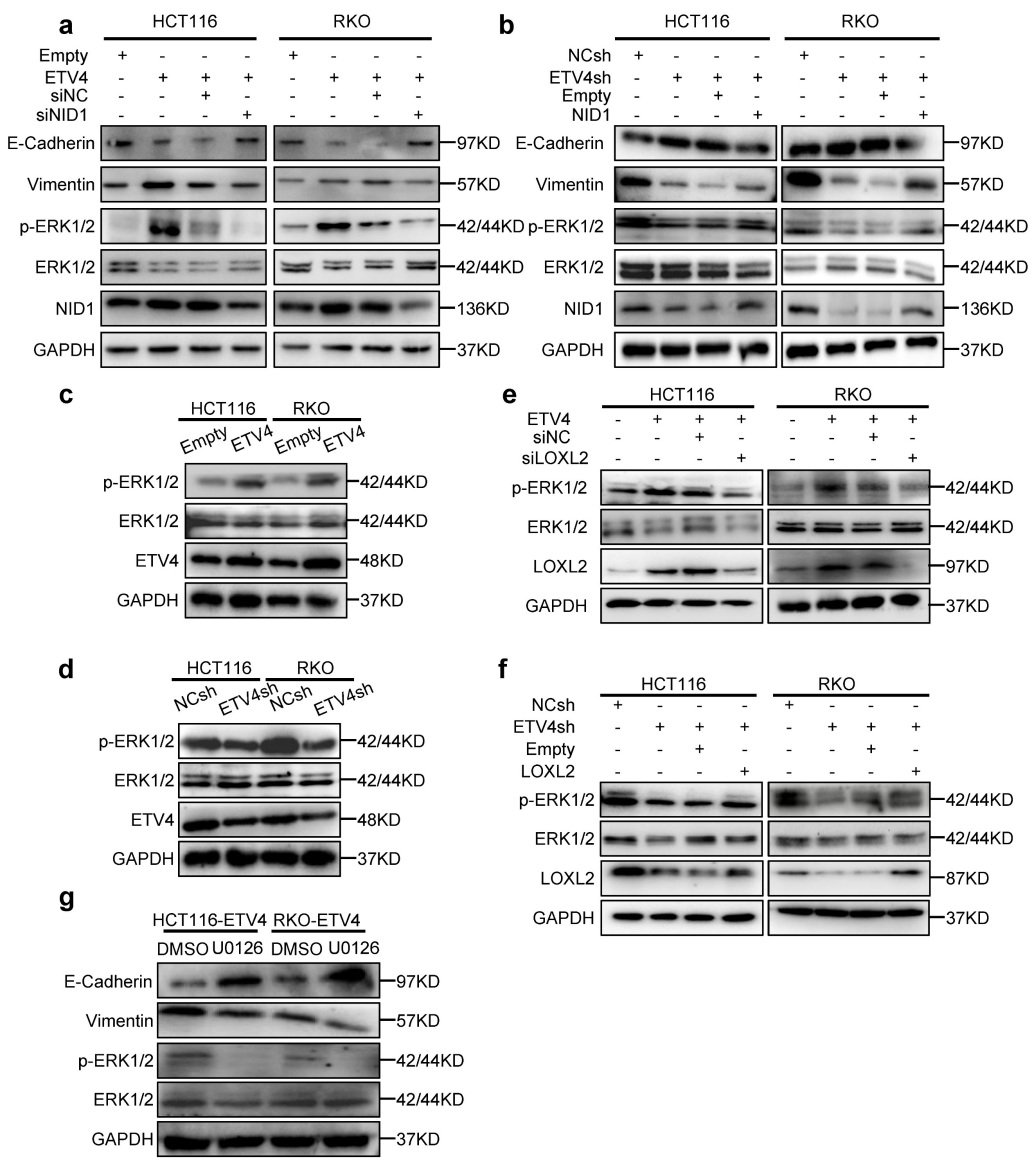
** ETV4/LOXL2/NID1 promotes EMT aggressive phenotype in CRC by activating ERK signaling pathway. a-b.** Silencing NID1 in stable ETV4 overexpression HCT116 and RKO cells, or upregulating NID1 in stable ETV4 knockdown HCT116 and RKO cells. The cell lysates were subjected to immunoblot analysis for detecting the expression of NID1, p-ERK, ERK, EMT markers. **c-d.** Immunoblotting was performed to determine ETV4, ERK1/2 and p-ERK1/2 in stable ETV4 overexpression and knockdown HCT116 and RKO cells. **e.** Silencing LOXL2 in stable ETV4 overexpression HCT116 and RKO cells, and immunoblotting was conducted to determine the protein expression of LOXL2, p-ERK and ERK. **f.** Overexpressing LOXL2 in stable ETV4 knockdown HCT116 and RKO cells, and immunoblotting was conducted to determine the protein expression level of LOXL2, p-ERK and ERK. **g.** Immunoblotting was performed to determine the protein expression of p-ERK, ERK, E-cadherin and Vimentin after U0126 (ERK inhibitor) treatment in stable ETV4 overexpression HCT116 and RKO cells.

Next, to interrogate the role of NID1 in ETV4-mediated CRC malignant phenotypes, CCK8 assay was performed to detect the cell viability when NID1 knockdown. As shown in Fig.6h-i and Fig.S14a-b, silencing NID1 weakened the proliferation enhanced by ETV4 overexpression in CRC cells while NID1 overexpression could restore the decreased cell proliferation in stable ETV4 knockdown cells. Moreover, the wound healing assay, transwell migration and invasion assay showed that silencing NID1 inhibited the ETV4-induced enhancement of cell migration and invasion, while NID1 overexpression rescued the ETV4sh-mediated inhibition of metastasis in different CRC cell lines (Fig.6j-k and Fig.S14c-e). Furthermore, immunoblot assay also demonstrated that silencing NID1 could exert the inhibited effect on EMT process enhanced by ETV4 (Fig.7a), but overexpression NID1 promoted the EMT repressed by ETV4 knockdown (Fig.7b). Taken together, NID1 is a pivotal downstream gene of ETV4/LOXL2 promoting aggressive phenotype in colorectal cancer.

### 3.7 LOXL2 and NID1 are required for ETV4-mediated activation of ERK signaling pathway in CRC cells

To further investigate the possible underlying mechanism about ETV4/LOXL2/NID1, we yielded the ERK-pathway-related gene set (BIOCARTA_ERK_PATHWAY) through the previous RNA-seq data (Fig.3b). Considering that ERK signaling pathway is the most significant signal activation pathway in colorectal cancer metastasis. We next to verify whether ETV4/LOXL2/NID1 induce EMT transformation and metastasis in colorectal cancer cells by activating ERK signaling pathway.

Firstly, we determined whether ETV4/LOXL2/NID1 signaling pathway could regulate ERK signaling pathway. As is shown in Fig.7c and 7d, we found that ETV4 overexpression increased phosphorylation of ERK1/2, while ETV4 knockdown resulted in a lower level of p-ERK1/2 in CRC cell lines. Next, we silenced LOXL2 in stable overexpression ETV4 CRC cells, and the results indicated that silencing LOXL2 weakened the phosphorylation of ERK1/2 induced by ETV4 (Fig.7e). Conversely, overexpressing LOXL2 enhanced the expression of p-ERK1/2 in ETV4sh-CRC cell lines (Fig.7f). Furthermore, we also found that NID1 knockdown attenuated the level of p-ERK1/2 in CRC cell lines stably overexpressed ETV4 (Fig.7a) while NID1 overexpression resulted in a higher level of p-ERK1/2 in ETV4sh-CRC cell lines (Fig.7b). Collectively, ETV4/LOXL2/NID1 could activate ERK signaling pathway.

In the previous study, we revealed that NID1 activates the ERK signaling pathway by autocrine activation of FAK in ovarian cancer^21^. And in this research, we found that ETV4 could activate the FAK signal pathway (Fig.S15a) and the FAK inhibitor (PF573228) repressed the phosphorylation of FAK and ERK1/2 induced by ETV4 (Fig.S15b). Furthermore, NID1 knockdown inhibited the phosphorylation of FAK (Fig.S15c). Taken together, our data indicated that ETV4/LOXL2/NID1 activates the ERK signaling pathway through FAK. Next, we explored whether ERK pathway is involved in ETV4-induced malignant phenotype. As shown in Fig.7g, the treatment of ERK inhibitor (U0126) not only decreased the level of p-ERK1/2 but also effected the expression of EMT markers in ETV4-overexpressed CRC cells. Taken together, the above results demonstrated that ETV4/LOXL2/NID1 induce EMT in CRC cells by activating ERK pathway.

### 3.8 ETV4 and LOXL2 induce NID1 transcription via demethylation of its promoter

Interestingly, NID1 promoter was highly methylated in colorectal cancer, and the differentially expressed gene in ETV4 overexpression RNA-seq data was enriched in the gene set MCGARVEY_SILENCED_BY_METHYLATION_IN_COLON_CANCER (Fig.3b), which indicates that ETV4 might regulate NID1 expression by mediating the methylation level of NID1 promoter. Further bioinformatics analysis via UCSC Xena database (https://xena.ucsc.edu/public/) also demonstrated that NID1 promoter methylation status was negatively correlated with ETV4 and LOXL2 (Fig.S16a). Consistently, the correlation analysis via TCGA database verified that the mRNA expression level of NID1 and LOXL2 was negative related with the promoter methylation of NID1 (Fig.S16b).

**Figure 8:**
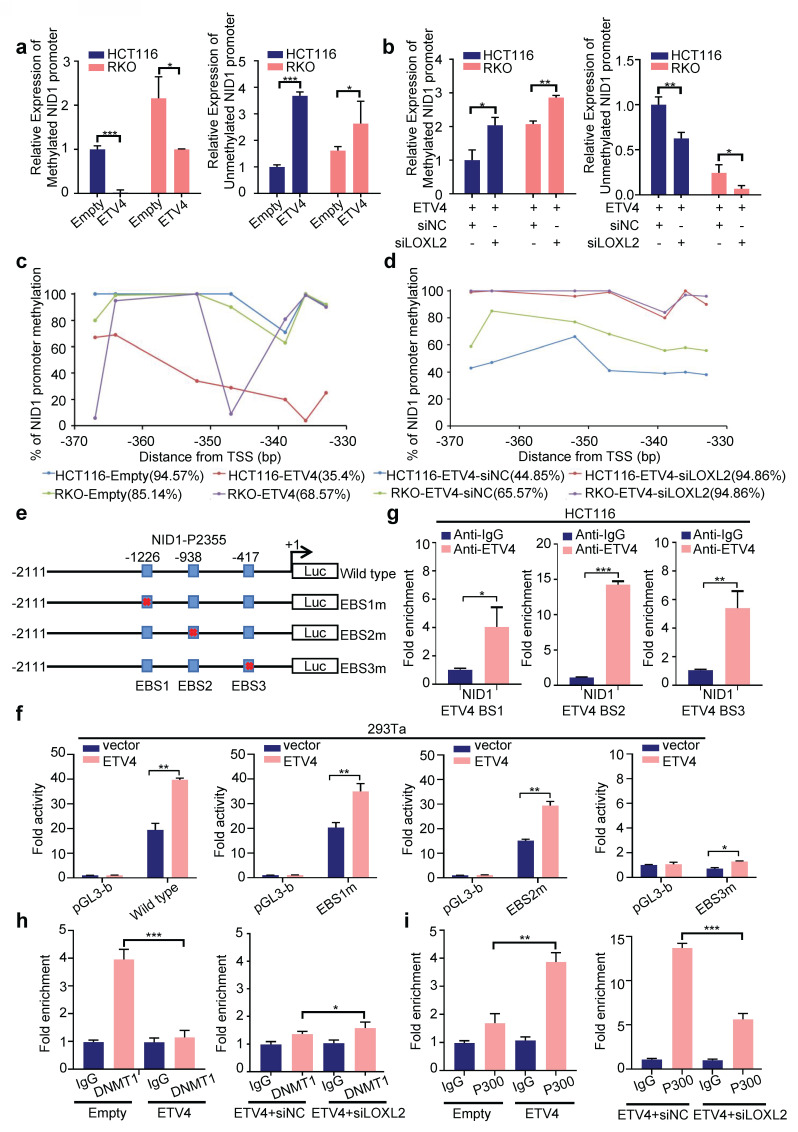
** ETV4 and LOXL2 promote NID1 transcription through promoter demethylation. a, c.** The methylation of NID1 promoter was determined by MSP or pyrosequencing in stable ETV4 overexpression HCT116 and RKO cells. **b, d.** Stable ETV4 overexpression HCT116 and RKO cells were transiently transfected with negative siRNA or LOXL2 siRNA. The methylation of NID1 promoter was determined by MSP or pyrosequencing in the indicated cells. **e.** Schematic illustration of the wildtype and mutant NID1-P2355 luciferase (Luc) promoter reporters. The transcription site for NID1 gene is indicated as +1. The ETV4 binding sites are shown as boxes. The mutated sites are crossed. **f.** Luciferase reporter assays. 293Ta cells were transiently transfected with the indicated plasmids, and forty-eight hours after transfection, the luciferase activities were measured. **g.** ChIP assay. Chromatin fragments were prepared from HCT116 cells and immunoprecipitated with anti-ETV4 antibody or control IgG. The precipitated DNA was then amplified by RT-PCR with primers directed to the ETV4 binding sites in the NID1 promoter region. **h-i**. ChIP assay. Chromatin fragments were prepared from HCT116 cells with stable ETV4 overexpression or further silencing LOXL2, and then immunoprecipitated with anti-DNMT1 antibody (h), anti-P300 (i) or control IgG. The precipitated DNA was then verified by RT-PCR. Data represent the mean ± SD. All experiments were performed in triplicates. *p < 0.05, **p < 0.01, *** p < 0.001.

To further reveal the association of NID1 promoter methylation with ETV4 and LOXL2, the methylation status of the NID1 promoter was firstly determined by methylation specific PCR (MSP), and the results showed that the promoter methylation level was impaired and the unmethylation level was increased in ETV4 overexpression group compared with the control group in HCT116 and RKO cells (Fig.8a and Fig.S16c). Furthermore, silencing LOXL2 could increase the NID1 promoter methylation level decreased by ETV4, while exert an inhibited effect on ETV4-induced elevation of NID1 promoter unmethylation levels (Fig.8b). Next, a total of 7 CpG sites located at nucleotides -370 and -330 in the NID1 promoter were examined by pyrosequencing (Fig.S16d). Consistent with MSP data, our pyrosequencing results also demonstrated that NID1 promoter was hypermethylated but ETV4 overexpression could decrease the methylated level (Fig.8c) and silencing LOXL2 would mitigate the decreased methylated level caused by ETV4 (Fig.8d). The correlation analysis also verified that these CpG sites (cg02454364, cg07578215, cg04204188, cg06094815) were negatively related with ETV4 and LOXL2 expression (Fig.S16e). Collectively, our results determined that ETV4 and LOXL2 could decrease the methylation of NID1 promoter.

We then analyzed the mechanism by which ETV4/LOXL2 mediates the demethylation of NID1 promoter. LOXL2 cannot directly bind to DNA, thus we firstly analyzed whether ETV4 can bind to the NID1 promoter. The transcription factor binding sites analyze via the Jasper database showed that there were three putative binding sites for ETV4 in NID1 promoter (Fig.8e and Fig.S17a). The NID1 promoter (-2111/+244) was cloned into the firefly luciferase reporter vector, and were co-transfected with pcDNA3.0-ETV4 into cells. The results indicated that ETV4 partly increased the luciferase activity in cells co-transfected with the NID1-P2355 (-2111/+244) compared with the control plasmid, suggesting that ETV4 could transactivate the NID1 promoter to some extent (Fig.8f). And ChIP assay determined that ETV4 could bind NID1 promoter region (Fig.8g). Following, the point mutations was performed at each ETV4 binding site within the NID1-P2355 (-2111/+244) region, respectively (Fig.8e). As shown in Fig.8f, the mutation of ETV4 binding site3 (EBS3m) could sharply decrease the NID1 luciferase activity, but the ETV4-mediated transactivation of NID1 promoter in other mutation was slightly weakened. Taken together, ETV4 could regulate expression of NID1 by transactivation, but ETV4-mediated methylation is the more important regulatory mechanism.

DNA demethylation occurs in rapidly dividing cells, usually caused by the inhibition of DNMT1 or by active removing the methyl group by TET. Thus, the mechanism of NID1 promoter demethylation was to investigated. The DAC (5-aza-2'-deoxycytidine, DNA methyltransferase inhibitor) treatment could reverse the hypermethylation of NID1 promoter (Fig.S17b) and enhance the expression level of NID1 in ETV4-siLOXL2 CRC cells (Fig.S17c-d), suggesting that DNMT (DNA methyltransferase) might mediate the methylation process of NID1 promoter. Next, the ChIP assay showed that ETV4/LOXL2 could inhibit DNMT1 binding to NID1 promoter (Fig.8h), but not affect TET1 binding activity (data not shown). And ChIP data also demonstrated that ETV4/LOXL2 enhanced P300 binding to the promoter of NID1 (Fig.8i). Collectively, our data indicated that ETV4/LOXL2 can induce NID1 transcriptional expression by inhibiting DMNT1 binding to the NID1 promoter region to demethylate, and recruiting P300 to promote acetylation.

### 3.9 ETV4 interacts with LOXL2 in CRC cells

**Figure 9:**
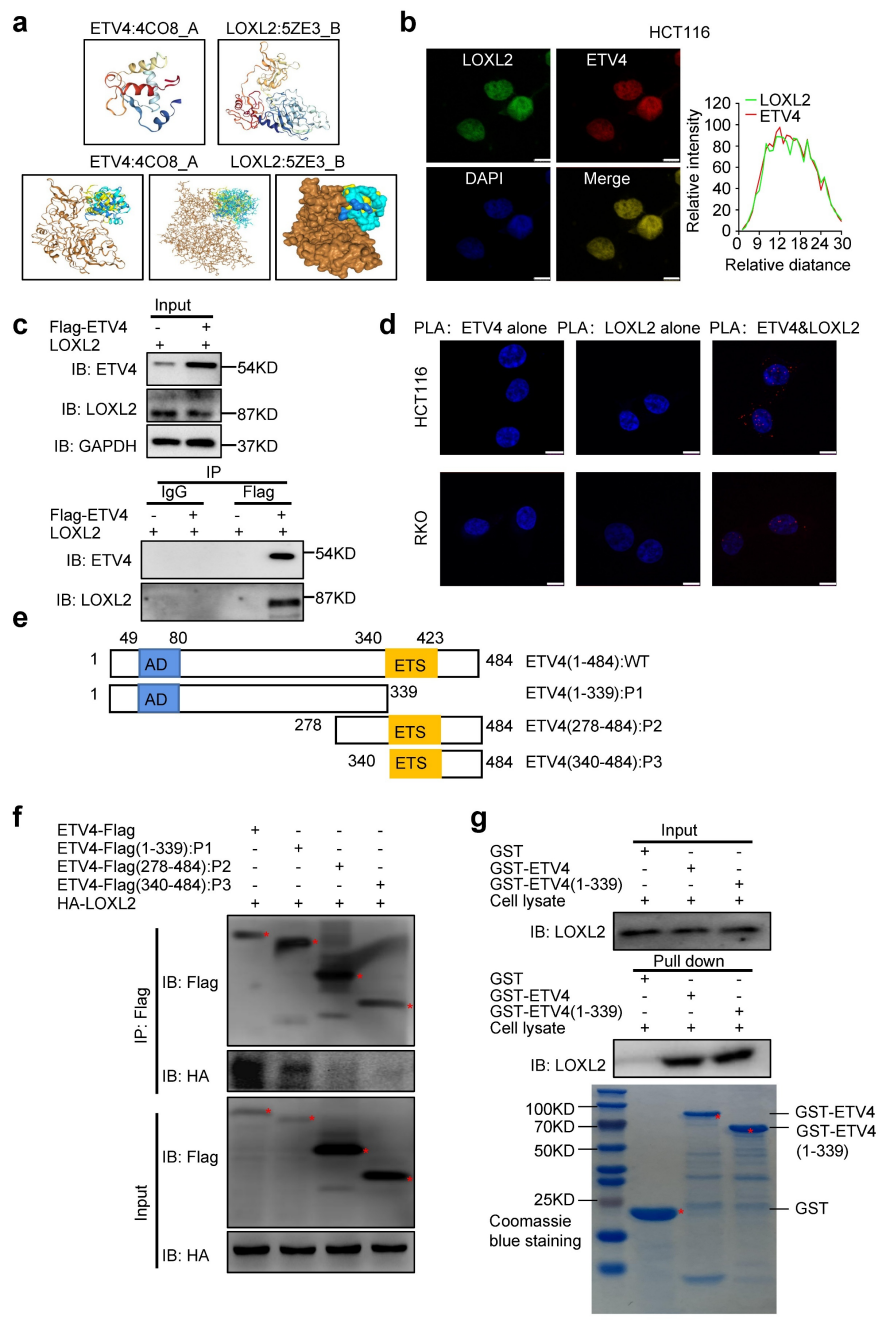
** Interaction between ETV4 and LOXL2. a.** Predicted models of ETV4-LOXL2 protein docking. The 3D homologous structures of ETV4 (PDB id: 4CO8) and LOXL2 (PDB id: 5ZE3) were searched by online ZDOCK server, and the three-dimensional docking models for ETV4 and LOXL2 interaction were predicted (ETV4 in color, LOXL2 in gold). **b.** Co-localization of ETV4 and LOXL2 protein expression in HCT116 cells nuclei. Representative images of immunofluorescence assay for ETV4 (red), LOXL2 (green) and DAPI (blue) in the indicated cells (left), scale bar: 8μm. Quantification for intensity of LOXL2 and ETV4 showing their colocalization (right). **c.** The interaction between ETV4 and LOXL2 was determined by co-immunoprecipitation assay in 293Ta cells co-transfected with pcDNA3.0-Flag-ETV4 and pcDNA3.1-HA-LOXL2 expression vector for 48h. **d.** Proximity ligation assays (PLAs) of ETV4 and LOXL2 in HCT116 and RKO cells. Red spots are regions of signal amplification. Nuclear stain (DAPI) is blue. Incubation with either ETV4 or LOXL2 antibody alone was used as a control. **e.** Schematic illustration of the ETV4 structure and deletion mutants. AD, acidic domain; ETS, ETS DNA binding domain. **f.** 293Ta cells were transiently co-transfected with pcDNA3.0-Flag-ETV4 or the indicated ETV4 deletion mutants with pcDNA3.1-HA-LOXL2 for 48h, and whole cell lysates were prepared and subjected to co-immunoprecipitation assay. **g.** GST pull-down assay. The whole cell lysates of HCT116-ETV4 cells, incubated with purified GST-ETV4 fusion proteins, GST-ETV4-P1(1-339AA) fusion proteins or GST-native proteins. And the products were detected with anti-LOXL2 antibody. GST and the purified lysates were indicated in Coomassie Blue staining.

According to our previous data, both ETV4 and LOXL2 regulate the transcription of NID1. ETV4 is a classic transcription factor and could mediate NID1 promoter demethylation of NID1 promoter by directly binding to the potential ETV4 binding site on NID1 promoter region. But, LOXL2 belongs to the lysyl oxidase (LOX) family of proteins, could not directly bind to NID1 promoter to produce its regulatory function. Thus, we assume that ETV4 and LOXL2 could interact and bind to the promoter region of NID1 to promote NID1 demethylation and transcription. To test this hypothesis, we firstly conducted a computational docking analysis of ETV4 and LOXL2 via the HDOCK server. The result showed that the highest-scored predicted docking model of interaction between ETV4 (PDB id: 4CO8) and LOXL2 (PDB id: 5ZE3) (Fig.9a). Then, immunofluorescence assay indicated that ETV4 co-localized with LOXL2 in CRC cell nuclei (Fig.9b). Moreover, the co-IP (co-immunoprecipitation) assay demonstrated that anti-Flag-ETV4 immunoprecipitants contained LOXL2, suggesting that ETV4 might form a complex with LOXL2 *in vivo* (Fig.9c). Next, we performed *in situ* PLA (proximity ligation assay), which can visualize protein interaction in close proximity (<40 nm) in cells. As shown in Fig.9d, ETV4 and LOXL2 were verified to locate in close proximity, as evidenced by the detected fluorescent PLA spots. Together, these results preliminarily confirmed that ETV4 could directly interact with LOXL2.

To further reveal the interaction between ETV4 and LOXL2, a series of truncation of ETV4 were conducted (Fig.9e), and were co-transfected respectively with pcDNA3.1-HA-LOXL2 to conduct the subsequent co-IP assay. As shown in Fig.9f, ETV4(1-339AA) retained the ability to bind LOXL2, whereas ETV4(278-484AA) and ETV4(340-484AA) did not. Next, we performed the GST pull-down assay *in vitro*. The whole cell lysates of HCT116-ETV4 cells, incubated with purified GST-ETV4 fusion proteins, GST-ETV4-P1(1-339AA) fusion proteins or GST-native proteins. Consistently with previous co-IP results, LOXL2 was pulled down by GST-ETV4 or GST-ETV4-P1 while not GST (Fig.9g). Altogether, these data demonstrated that the interaction of ETV4 with LOXL2 might be mediated at least by ETV4 N-terminal regions (1-339AA).

## 4. Discussion

Colorectal cancer (CRC) is one of the malignant tumors of the digestive system, and its metastasis led to the death of thousands every year^38^. In this study, we demonstrated that the expression of ETV4 was remarkably upregulated in CRC tissues and significantly related to the lymph node metastasis and the stage of cancer. Functional study revealed that ETV4 promoted the cell growth, EMT and metastasis in CRC. Mechanistically, ETV4 increased the expression levels of LOXL2 by activating its transcription and LOXL2 was vital for ETV4-mediated malignant phenotypes in CRC. Meanwhile, ETV4 could recruit LOXL2 to the promoter region of NID1 for mediating demethylation and expression of NID1, which in turn activated the ERK pathway to promote cell proliferation, EMT and metastasis in CRC. Taken together, our study indicated that a vital role of the novel ETV4/LOXL2/NID1 axis in CRC progression.

ETV4, as a canonical transcription factor, could mediate the development and progression in multiple cancers through transcriptionally regulating its targeted genes, such as Cyclin D1, CXCR4, ANXA2 and KDM5D^39^-^42^. For example, in pancreatic ductal adenocarcinoma (PDAC), ETV4 was demonstrated to be remarkably upregulated in tumor tissues compared with the normal tissues and could promote the cell growth by transcriptionally regulating Cyclin D1^39^. Xu *et al.* demonstrated that ETV4 promoted tumor metastasis by transcriptionally activating FOSL1 with a PI3K/AKT dependent manner in clear cell renal cell carcinoma^43^. Consistently, ETV4 has been reported to promote CRC progression by regulating the expression of MMP1, HES1 and STAT3^44^, ^45^. These findings indicated that ETV4 may serve as a therapy target in various cancers. For example, Kim *et al.* found that CIC, a transcription repressor, could inhibit HCC progression by targeting ETV4^46^. Another study of Leng *et al.* demonstrated that ETV4 was repressed by miR-29b, thereby inactivating the ERK signaling pathway and inhibiting EMT in CRC^47^. And beyond that, ETV4 has been found to synergistically transcribe with other partners. For example, Wollenick *et al.* found that in hypoxic conditions, ETV4 could cooperate with HIF-1α through binding to the CAD of HIF-1α, thereby transactivating the targeted genes, such as PHD2^48^. In addition, ETV4 has also been established as a vital influencer in chromatin accessibility^9^, ^49^. For instance, it is reported that ETV4 served as a necessary pioneer factor and affected chromatin accessibility by a small subset of ER binding site and ETV4 also impacted ER nuclear location, thereby affected estrogen signaling in endometrial cancer^9^. Moreover, Arase *et al.* found that silencing ETV4 inhibited the TGF-β-mediated expression of MMP13 and the cell invasion by reducing the chromatin accessibility at the enhancer region of MMP13 gene in mammary gland epithelial cells^49^. Taken together, ETV4 could be a promising target of cancer therapy as a transcription factor, coactivator or chromatin modulator.

In the present study, we found that LOXL2 was a direct targeted gene of ETV4 and essential for ETV4-mediated malignant phenotypes in CRC. LOXL2 is one of the lysyl oxidase (LOX) family members which serves as an amine oxidase depending on the copper and lysine tyrosyl-quinone^50^. In recent years, LOXL2 has been reported to act as a multifunction enzyme involved in mediating numerous cellular processes in cancers. For example, LOXL2 was found to promote the ECM remodeling by enhancing the expression and activity of TIMP1 and MMP9, thereby leading to metastasis in breast cancer^51^. Nguyen *et al.* pointed out that CAF-derived LOXL2 produced a highly aligned ECM by autocrine and paracrine signaling in tumor microenvironment in prostate cancer, thereby promoting the migration of CAF and neighbored prostate tumor cells^52^. Furthermore, Zhan *et al.* demonstrated that LOXL2 promoted the esophageal squamous cell carcinoma (ESCC) metastasis by inducing ezrin phosphorylation-mediated cytoskeletal reorganization^53^. Besides, LOXL2 plays vital intranuclear roles in transcriptional regulation among various cancers^54^. For instance, a study revealed that LOXL2 interacted with Snail and synergistically repressed the expression of E-cadherin, thereby induced EMT process^55^. Li *et al.* demonstrated that LOXL2 could reciprocally regulate with HIF1α to drive the Warburg effect for mediating the aggressive phenotypes in pancreatic cancer^14^. In addition, increasing study underlined the role of LOXL2 in epigenetic regulation. For example, Herranz *et al.* revealed that LOXL2 could catalyze the trimethylated H3K4 deamination by generating another type of H3, an oxidized H3, which represented an unconventional chemical modification of H3K4^56^. For the first time, our study demonstrated that ETV4 overexpression could transactivate LOXL2 and recruit it to promoter region of NID1 for mediating promoter demethylation and expression of NID1.

NID1, a member of the extracellular matrix binding protein nidogen family, is a secretory glycoprotein and an important component of the basal membrane^21^. An increasing number of evidences have indicated that NID1 was hypermethylation in cancers, such as breast cancer, gastrointestinal cancer and colorectal cancer^22^, ^57^, ^58^. Several recent reports found that the overexpression of NID1 may contribute to the progression of some cancers^25^, ^59^, ^60^. For example, Zhou *et al.* found that NID1 was overexpression in gastric cancer tissues and related with the poor outcome, and NID1 promoted the cell migration and invasion by EMT in gastric cancer^59^. Specifically, NID1, secreted from extracellular vesicles (EVs) in metastatic HCC enhanced the pre-metastatic niche formation in lung through increasing angiogenesis and the pulmonary endothelial permeability, thereby promoting tumor cells colonization and extrahepatic metastasis^60^. In CRC, Rokavec *et al.* revealed that colorectal cancer cells accelerated the tumor progression by secreting NID1, which thereby mediated EMT in neighbor tumor cells^58^. These findings strongly suggest that NID1 has essential function on the development of cancers. In addition, Gaggero *et al.* pointed that NID1 could also serve as the ligand of NKp44 which is an essential activating receptor expressed in activated NK cells, to activate NK cell function via regulating NKp44-mediated IFN-γ production or cytotoxicity^61^, which indicating NID1 may be involved in immunotherapy of solid tumors. Intriguingly, Pedrola *et al.* found that NID1 was transcriptionally regulated by ETV5, the close homologs of ETV4, suggesting PEA3 family members could regulate NID1 expression by binding its ETS site^23^. And in the present study, we found that NID1 could be epigenetically activated by ETV4 and significant for ETV4-induced aggressive phenotypes in CRC by activating ERK signaling pathway. Consistent with our study, Zhou *et al.* demonstrated that NID1 could activate FAK/ERK pathway to induce the cell migration, invasion, chemoresistance and EMT process in ovarian cancer^21^. More interestingly, some previous studies have verified that the expression of ETV4 could also be enhanced by ERK pathway in cancers. For example, Tan *et al.* revealed that p-ERK molecule enhanced the expression of ETV4 by repressing ETV4 proteasomal degradation to promote invasion, metastasis and worse prognosis in colorectal mucinous adenocarcinoma (CRMAC)^62^. Thus, there may be exhibit an exquisite mutual collaboration and reciprocal cross regulation between ETV4/LOXL2/NID1 axis and ERK signaling in the malignant progression of CRC.

In summary, we revealed that LOXL2 was a novel targeted gene of ETV4 and directly interacting partner of ETV4, which was important for ETV4-induced CRC malignant phenotypes. In addition, ETV4 interacting with LOXL2 could synergistically promote the expression of targeted genes such as NID1 through mediating DNA methylation, which will further lead to the cell proliferation and metastasis of CRC by the activation of ERK signaling pathway. This study provides a molecular basis to understand the underlying mechanisms of metastasis in CRC, and the novel ETV4/LOXL2/NID1 axis may be useful to develop the new strategies to treat those patients with CRC.

## 5. Conclusions

**Figure 10:**
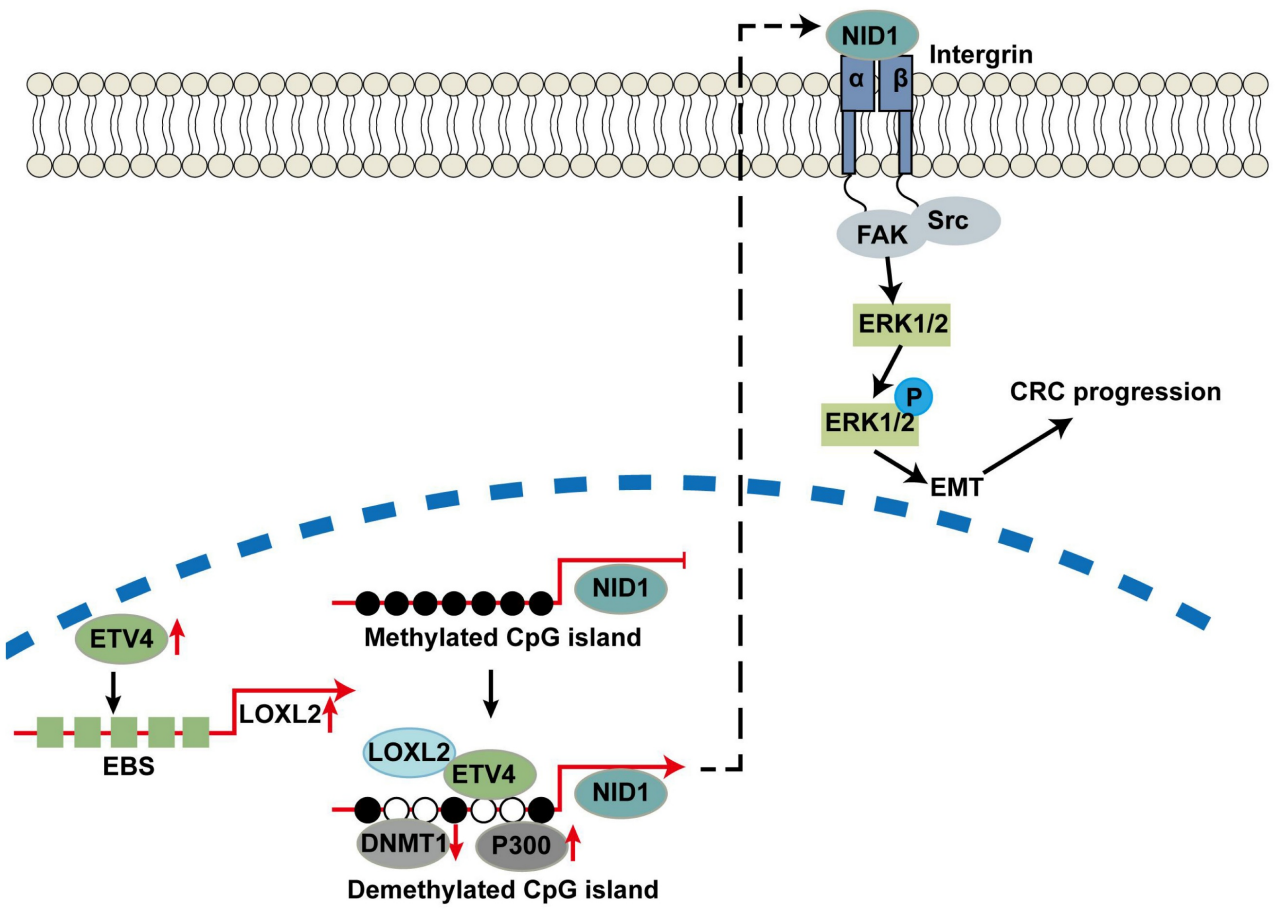
** Work model.** ETV4 could transactivate LOXL2 and recruit it to the promoter of NID1 to mediate the promoter demethylation and expression of NID1, which in turn activates ERK pathway and promotes CRC metastasis.

In summary, we revealed that LOXL2 was a novel targeted gene of ETV4 and directly interacting partner of ETV4, which was important for ETV4-induced CRC malignant phenotypes. In addition, ETV4 interacting with LOXL2 could synergistically promote the expression of targeted genes such as NID1 through mediating DNA methylation, which will further lead to the cell proliferation and metastasis of CRC by the activation of ERK signaling pathway (Fig.10). This study provides a molecular basis to understand the underlying mechanisms of metastasis in CRC, and the novel ETV4/LOXL2/NID1 axis may be useful to develop the new strategies to treat those patients with CRC.

## Supplementary Material

Supplementary figures.

Supplementary tables.

Supplementary file 1.

Supplementary file 2.

Supplementary file 3.

## Abbreviations

CRC: colorectal cancer
EMT: epithelial-mesenchymal transition
GST: glutathione-S-transferase
ETV4: ETS variant 4
LOXL2: Lysyl oxidase-like 2
NID1: nidogen 1
ERK: extracellular regulated MAP kinase
FAK: focal adhesion kinase
TF: transcription factors
DEGs: differential expression genes
CDH1: cadherin 1
CDH2: cadherin 2
DNMT1: DNA methyltransferase 1
TET: Ten-Eleven Translocation
DAC: 5-aza-2'-deoxycytidine
MMP: matrix metalloproteinase
BM: basal membrane
GEPIA: Gene Expression Profiling Interactive Analysis
HPA: Human Protein Atlas
