# Supplementary material for: ETV4 interacts with LOXL2 to induce epigenetic activation of NID1 during colorectal cancer progression
Source: Int J Biol Sci. 2025 Oct 20;21(15):6674–96. doi: 10.7150/ijbs.116383 (PMC12631116; doi:10.7150/ijbs.116383)

Supplementary Figure Legends

Supplementary Figure 1. The differential expression genes (DEGs) of CRC in three datasets.

a. Workflow diagram for screening the potential tumor markers via different databases**.** **b-d.** Heat maps of differentially expression genes in three CRC datasets (GSE4183, GSE20916 and TCGA-COAD).

Supplementary Figure 2. The Human Protein Atlas analysis.

The protein expression summary analysis for ETV4 via the Human Protein Atlas.

Supplementary Figure 3. ETV4 enhanced cell proliferation and metastasis in CRC

a-b. Lentivirus-mediated stable ETV4 overexpression or knockdown HCT116, RKO and HT29 cells were selected in the presence of puromycin for 72h. Expression of ETV4 was examined by immunoblot analysis. **c.** Cell viability was determined by CCK8 assays in stable ETV4 overexpression or knockdown HT29 cells. **d.** Cell proliferation was analyzed by colony formation assay in HT29 cells with stable overexpression or knockdown ETV4. Cells were seeded into 6-well plates for two weeks and colony numbers were quantified. Representative images (left) and quantification (right) of colony formation of HT29 cells. **e-f.** Transwell migration and invasion assay of HT29 cells stable overexpression or knockdown ETV4. Representative images (left, scale bar: 100μm) and relative number of migrated or invaded cells (right). **g-h.** Stable ETV4 overexpression and knockdown HCT116, RKO and HT29 were performed to the wound healing assay. Data represent the mean ± SD. All experiments were performed in triplicates. *p < 0.05, **p < 0.01, *** p < 0.001.

Supplementary Figure 4. ETV4 overexpression was correlated with high proliferation of CRC in vivo

a, c. Stable ETV4 overexpression or knockdown HCT116 cells were injected subcutaneously into nude mice, then the expression level of ETV4 and PCNA was detected by immunoblot analysis in the indicated xenograft samples. **b, d.** IHC staining and H&E staining of the indicated xenograft samples as described in (a, c), scale bar: 50μm.

Supplementary Figure 5. ETV4 promotes EMT in colorectal cancer cells

a. Gene set enrichment analysis of RNA-seq results showed the enrichment of CDH1 in ETV4 overexpression HCT116 cells. **b-c.** Verification of the expression of E-cadherin, N-cadherin, Snail, Twist1 and Vimentin using RT-qPCR in HT29 cells with stable ETV4 overexpression or knockdown. **d.** Immunoblot analysis of E-cadherin, N-cadherin, Vimentin and Twist1 expression in HT29 cells stable ETV4 overexpression or knockdown. **e.** Immunofluorescence analysis for the expression levels of E-cadherin and Vimentin in stable ETV4 overexpression or knockdown HT29 cells, scale bar: 25μm. Data represent the mean ± SD. All experiments were performed in triplicates. $*p < 0.05$, $**p < 0.01$, $*** p < 0.001$.

Supplementary Figure 6. LOXL2 is upregulated and correlated with ETV4 in CRC

a. The LOXL2 mRNA expression were analyzed online in TCGA-COAD, GSE4183 and GSE20916 data sets. **b.** The mRNA expression of LOXL2 was detected via RT-qPCR in CRC cDNA array purchased from OriGene. **c-e.** Kaplan–Meier survival curve

analysis representing probabilities of disease-free survival in CRC patients based on the individual or combined expression of ETV4 and LOXL2. **f.** The nucleotide sequences of putative ETV4 binding sites are underlined within LOXL2 promoter. **g.** PCR analysis of ChIP. Chromatin fragments were prepared from HCT116 cells and immunoprecipitated with anti-ETV4 antibody or control IgG. The precipitated DNA was then amplified by real-time PCR with primers directed to the ETV4 binding sites in the LOXL2 promoter region.

Supplementary Figure 7. LOXL2 promotes cell proliferation, migration and invasion in CRC cells

a-b. HCT116 and RKO cells were transiently transfected with negative control siRNA (siNC) and siRNA against LOXL2 (siLOXL2). After 48h, RT-qPCR (a) and immunoblot analysis (b) were conducted to detect LOXL2 expression. **c-f.** HCT116 and RKO cells were treated as described in (a). Cell proliferation and motility were detected by CCK8 assay (c), transwell migration and invasion assay (d, scale bar: 100μm), and wound healing assay (e). Immunoblotting assay were conducted to determine the expression of E-cadherin, Vimentin and Twist1 (f). Data represent the mean ± SD. All experiments were performed in triplicates. $*p < 0.05$, $**p < 0.01$, $***\ p < 0.001$.

Supplementary Figure 8. Silencing LOXL2 in ETV4-overexpression CRC cells

a-b. siRNA mediated silencing of LOXL2 in stable ETV4 overexpression HCT116 and RKO cells. The LOXL2 expression was detect by RT-qPCR (a) and immunoblot analysis (b).

Supplementary Figure 9. LOXL2 is required for ETV4-induced malignant

phenotypes in CRC cells

a, **c, d, f, h.** Silencing of LOXL2 in stable ETV4 overexpression RKO and HCT116 cells. Cells were subjected to CCK8 assay (a), colony foramtion assay (c), transwell migration and invasion assay (d, scale bar: 100μm), and wound healing assay (f, h). **b, e, g, i.** Stable ETV4 knockdown CRC cells were transiently transfected with pcDNA3.1 empty or pcDNA3.1-HA-LOXL2 expression constructs. Then cell proliferation and motility were determined by CCK8 assay (b), transwell migration and invasion assay (e, scale bar: 100μm), and wound healing assay (g, i). **j.** Immunoblot analysis of E-cadherin, N-cadherin and Twist1 expression in RKO cells treated as described in (a). Data represent the mean ± SD. All experiments were performed in triplicates. *$p < 0.05$, **$p < 0.01$.

Supplementary Figure 10. LOXL2 regulated NID1 expression in CRC cells.

a-b. HCT116 and RKO cells were transiently transfected with pcDNA3.1 empty or pcDNA3.1-HA-LOXL2 expression constructs. After 48h, RT-qPCR (a) and immunoblot analysis (b) was conducted to detect NID1 expression.

Supplementary Figure 11. NID1 is one of the 15 nearest neighbors with LOXL2 based on tissue protein expression

Expression clustering & correlation for LOXL2 via the Human Protein Atlas database.

Supplementary Figure 12. NID1 is upregulated and may serve as a potential prognostic marker in CRC.

a. The NID1 mRNA expression were analyzed online in three CRC datasets (TCGA-COAD, GSE4183 and GSE20916). **b-e**. Kaplan–Meier survival curve analysis

representing probabilities of disease-free survival in CRC patients based on NID1 (b), as well as the combined expression of ETV4 and NID1 (c), LOXL2 and NID1 (d), and the triple combination of ETV4, LOXL2 and NID1 (e). **f.** Determination of association between EMT markers and NID1 via GSE4183 and GSE20916.

Supplementary Figure 13. NID1 play promoted role in cell growth, migration and invasion in CRC cells.

a-b. HCT116 and RKO cells were transiently transfected with negative control siRNA (siNC) and siRNA against NID1 (siNID1). After 48h, RT-qPCR (a) and immunoblot analysis (b) were conducted to detect NID1 expression. **c-f.** HCT116 and RKO cells were treated as described in (a). Cell proliferation and motility were detected by CCK8 assay (c), colony formation assay (d), transwell migration and invasion assay (e, scale bar: 100μm) and wound healing assay (f). Data represent the mean ± SD. All experiments were performed in triplicates. $^{*}p < 0.05$, $^{**}p < 0.01$, $^{***}p < 0.001$.

Supplementary Figure 14. NID1 is essential for ETV4-mediated aggressive phenotypes in CRC cells.

a-b,c,e. Stable ETV4 overexpression RKO cells were transiently transfected with negative siRNA or NID1 siRNA, or stable ETV4 knockdown RKO cells were transiently transfected with empty or pcDNA3.0-NID1. The cell viability and motility were determined by CCK8 assay (a-b), wound healing assay (c), transwell migration and invasion assay (e, scale bar: 100μm). **d.** Stable ETV4 overexpression HCT116 cells were transiently transfected with negative siRNA or NID1 siRNA, then cells were subjected to wound healing assay. Data represent the mean ± SD. All experiments were

performed in triplicates. **p < 0.01, *** p < 0.001.

Supplementary Figure 15. ETV4/LOXL2/NID1 activates the ERK signaling pathway through FAK

a. Immunoblotting was performed to determine FAK and p-FAK in stable ETV4 overexpression HCT116 and RKO cells. **b.** Immunoblotting was performed to determine the protein expression of p-ERK, ERK, p-FAK and FAK after PF573228 (FAK inhibitor) treatment in stable ETV4 overexpression HCT116. **c.** Immunoblotting was performed to explore the protein expression of p-FAK and FAK in HCT116 and RKO cells transiently transfected with negative siRNA or NID1 siRNA.

Supplementary Figure 16. ETV4 and LOXL2 promote NID1 transcription through promoter demethylation

a. The association of NID1 promoter methylation status with ETV4 and LOXL2 via UCSC Xena database (https://xena.ucsc.edu/public/). **b.** The association of NID1 methylation with NID1 or LOXL2 expression via TCGA database. **c.** Verification of NID1 promoter methylation status in stable ETV4 overexpression HCT116 and RKO cells using methylation specific PCR. **d.** Schematic illustration of 7 CpG sites located between nucleotides -370 and -330 in the NID1 promoter. **e.** The correlation of CpG sites in NID1 promoter region with ETV4 and LOXL2 via TCGA databases.

Supplementary Figure 17. ETV4/LOXL2 can induce NID1 transcriptional expression by promoter demethylation

a. The nucleotide sequences of putative ETV4 binding sites are underlined within NID1 promoter. **b.** Stable ETV4 overexpression HCT116 and RKO cells were transiently

transfected with negative siRNA or LOXL2 siRNA, and cells were treated with DAC (5-aza-2'-deoxycytidine, DNA methyltransferase inhibitor), then the methylation level of NID1 promoter was determined by MSP. **c-d.** HCT116 and RKO cells were treated as described in (**b**), then the expression of NID1 was determined by RT-qPCR (c) and immunoblot analysis (d). Data represent the mean ± SD. All experiments were performed in triplicates. **$p < 0.01$, *** $p < 0.001$.

Figure S1

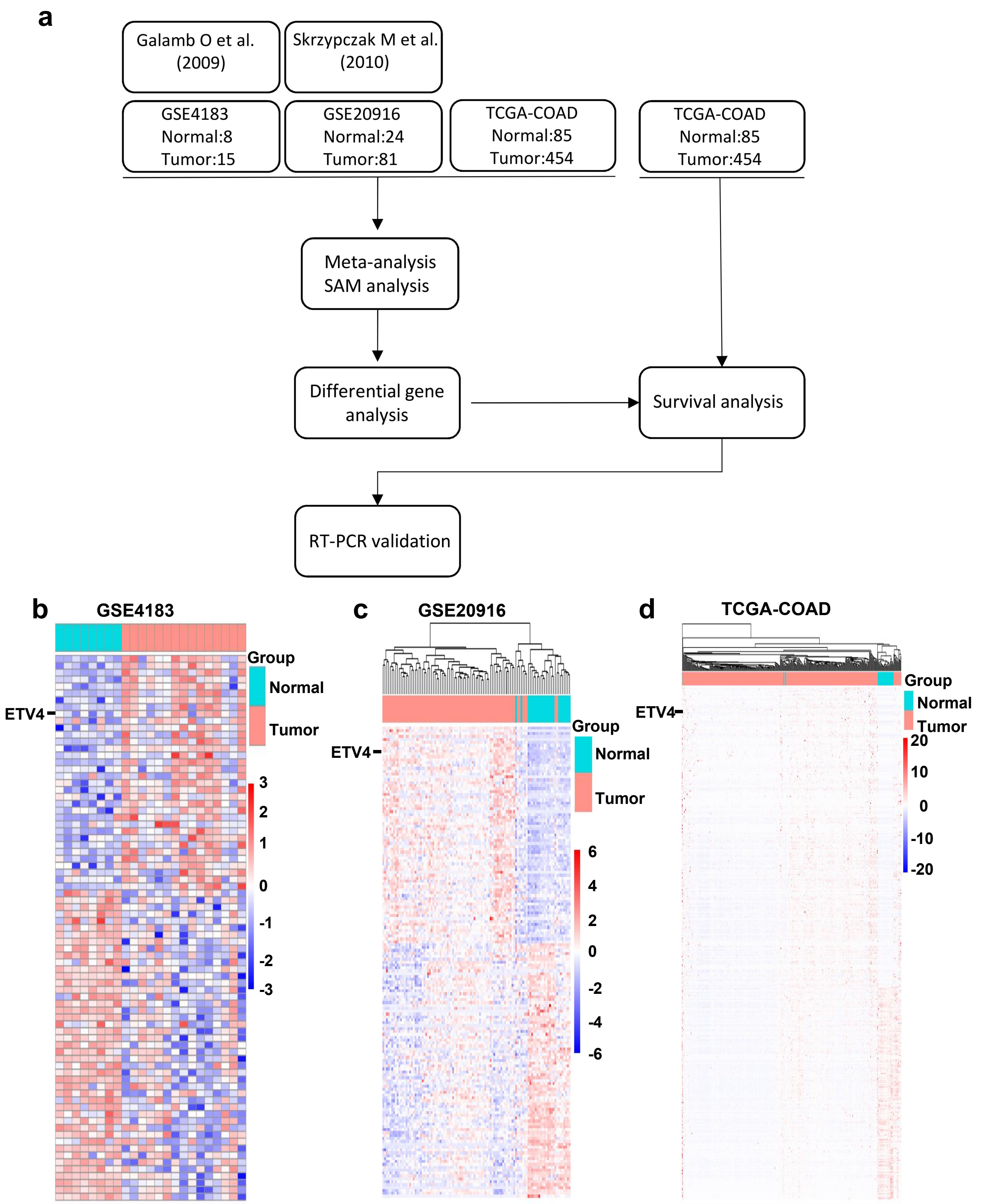
a
Galamb O et al. (2009)
Skrzypczak M et al. (2010)
GSE4183 Normal:8 Tumor:15
GSE20916 Normal:24 Tumor:81
TCGA-COAD Normal:85 Tumor:454
TCGA-COAD Normal:85 Tumor:454
Meta-analysis SAM analysis
Differential gene analysis
Survival analysis
RT-PCR validation
b
GSE4183
ETV4
Group
Normal
Tumor
3
2
1
0
-1
-2
-3
c
GSE20916
ETV4
Group
Normal
Tumor
6
4
2
0
-2
-4
-6
d
TCGA-COAD
ETV4
Group
Normal
Tumor
20
10
0
-10
-20

Figure S2

a

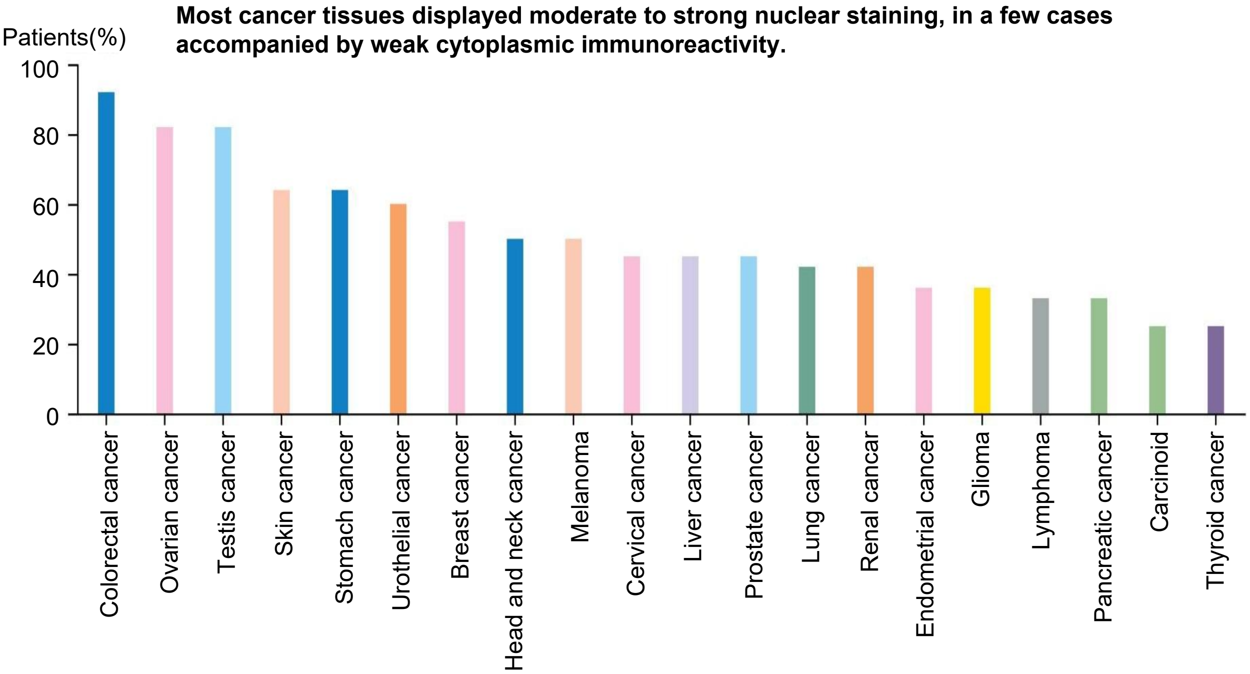

Most cancer tissues displayed moderate to strong nuclear staining, in a few cases accompanied by weak cytoplasmic immunoreactivity.
Patients(%)
100
80
60
40
20
0
Colorectal cancer
Ovarian cancer
Testis cancer
Skin cancer
Stomach cancer
Urothelial cancer
Breast cancer
Head and neck cancer
Melanoma
Cervical cancer
Liver cancer
Prostate cancer
Lung cancer
Renal cancar
Endometrial cancer
Glioma
Lymphoma
Pancreatic cancer
Carcinoid
Thyroid cancer

Figure S3

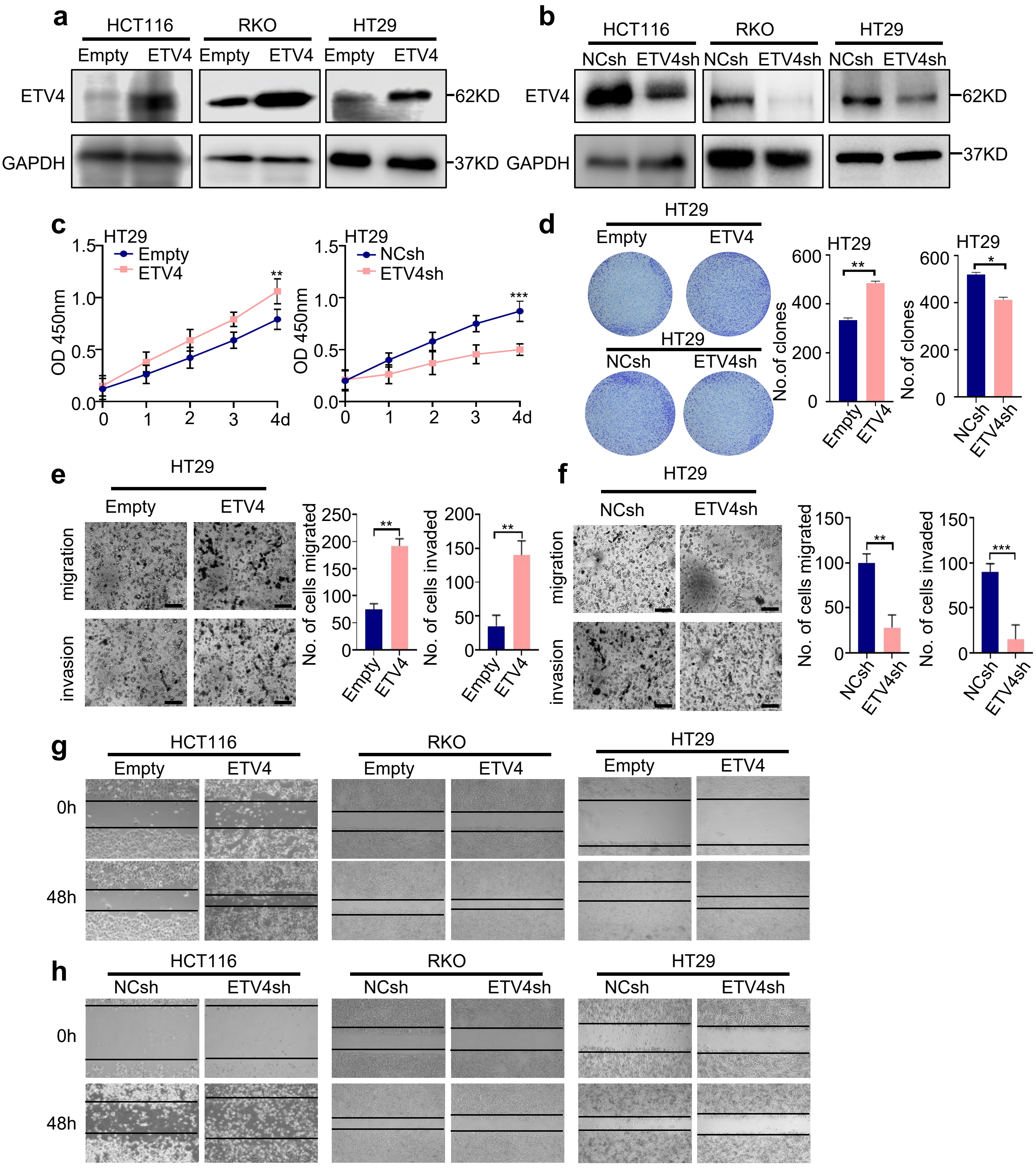
a
HCT116
RKO
HT29
Empty
ETV4
ETV4
GAPDH
62KD
37KD
b
NCsh
ETV4sh
c
OD 450nm
Empty
ETV4
NCsh
ETV4sh
4d
d
No.of clones
e
HT29
migration
invasion
No. of cells migrated
No. of cells invaded
f
g
0h
48h
h

Figure S4

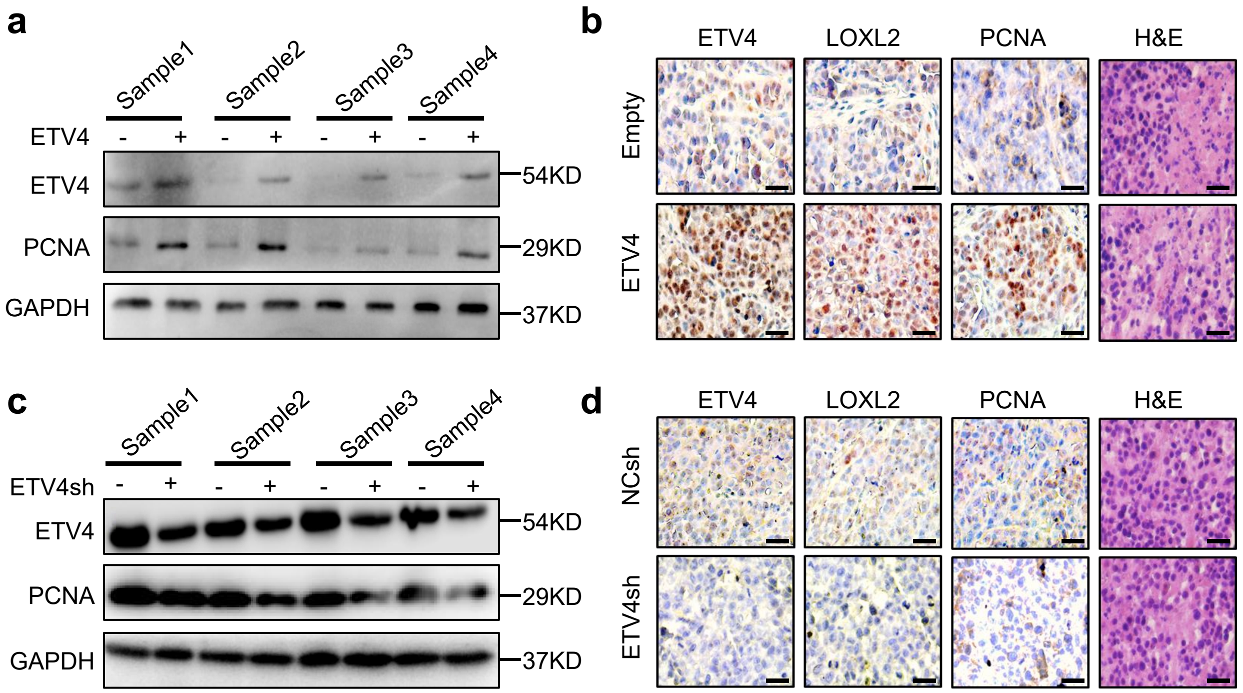

a
Sample1
Sample2
Sample3
Sample4
ETV4
- + - + - + - +
ETV4
PCNA
GAPDH
54KD
29KD
37KD
b
ETV4
LOXL2
PCNA
H&E
Empty
ETV4
c
Sample1
Sample2
Sample3
Sample4
ETV4sh
- + - + - + - +
ETV4
PCNA
GAPDH
54KD
29KD
37KD
d
ETV4
LOXL2
PCNA
H&E
NCsh
ETV4sh

Figure S5

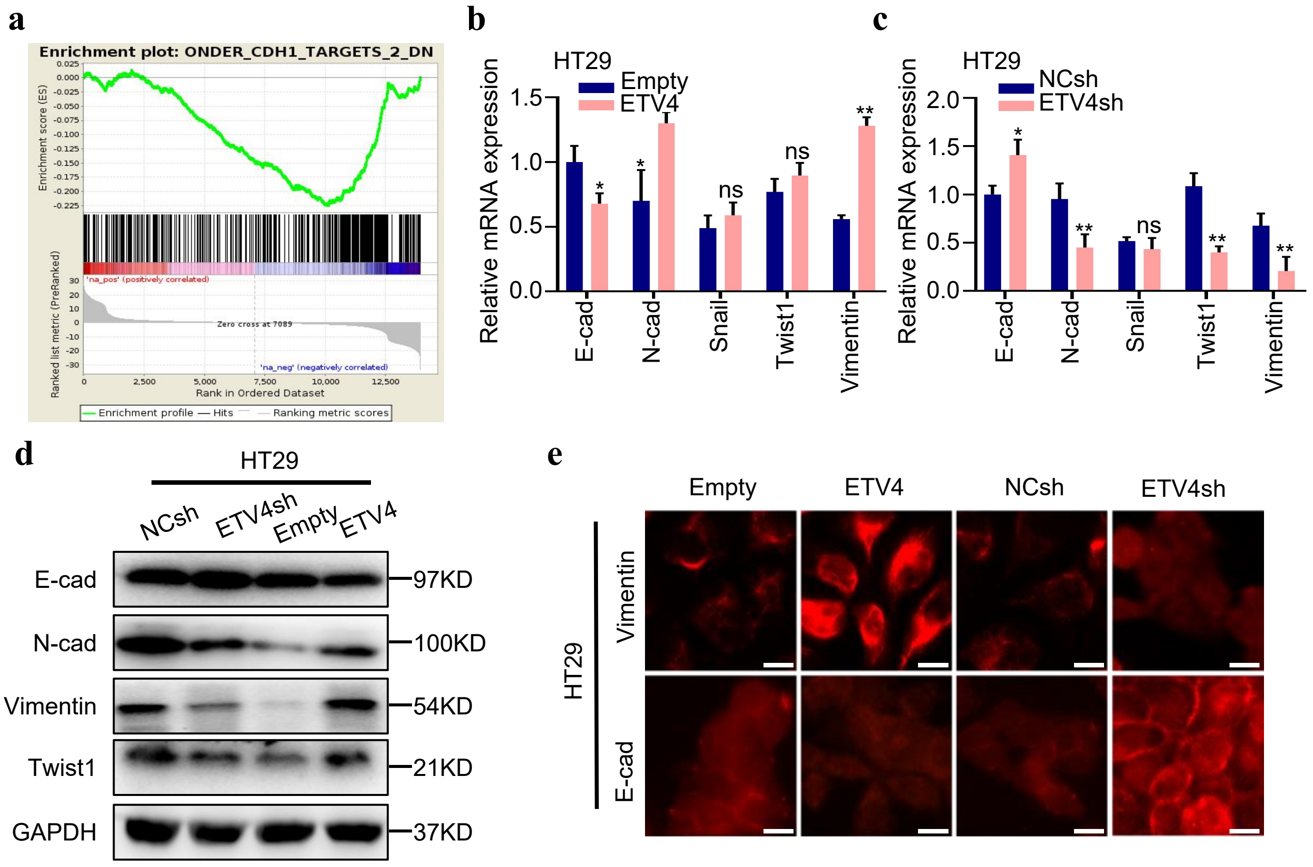

a
Enrichment plot: ONDER_CDH1_TARGETS_2_DN
Enrichment score (ES)
Ranked list metric (PreRanked)
'na_pos' (positively correlated)
Zero cross at 7089
'na_neg' (negatively correlated)
Rank in Ordered Dataset
Enrichment profile — Hits — Ranking metric scores
b
HT29
Empty
ETV4
Relative mRNA expression
E-cad
N-cad
Snail
Twist1
Vimentin
ns
c
HT29
NCsh
ETV4sh
Relative mRNA expression
E-cad
N-cad
Snail
Twist1
Vimentin
ns
d
HT29
NCsh
ETV4sh
Empty
ETV4
E-cad
97KD
N-cad
100KD
Vimentin
54KD
Twist1
21KD
GAPDH
37KD
e
Empty
ETV4
NCsh
ETV4sh
HT29
Vimentin
E-cad

Figure S6

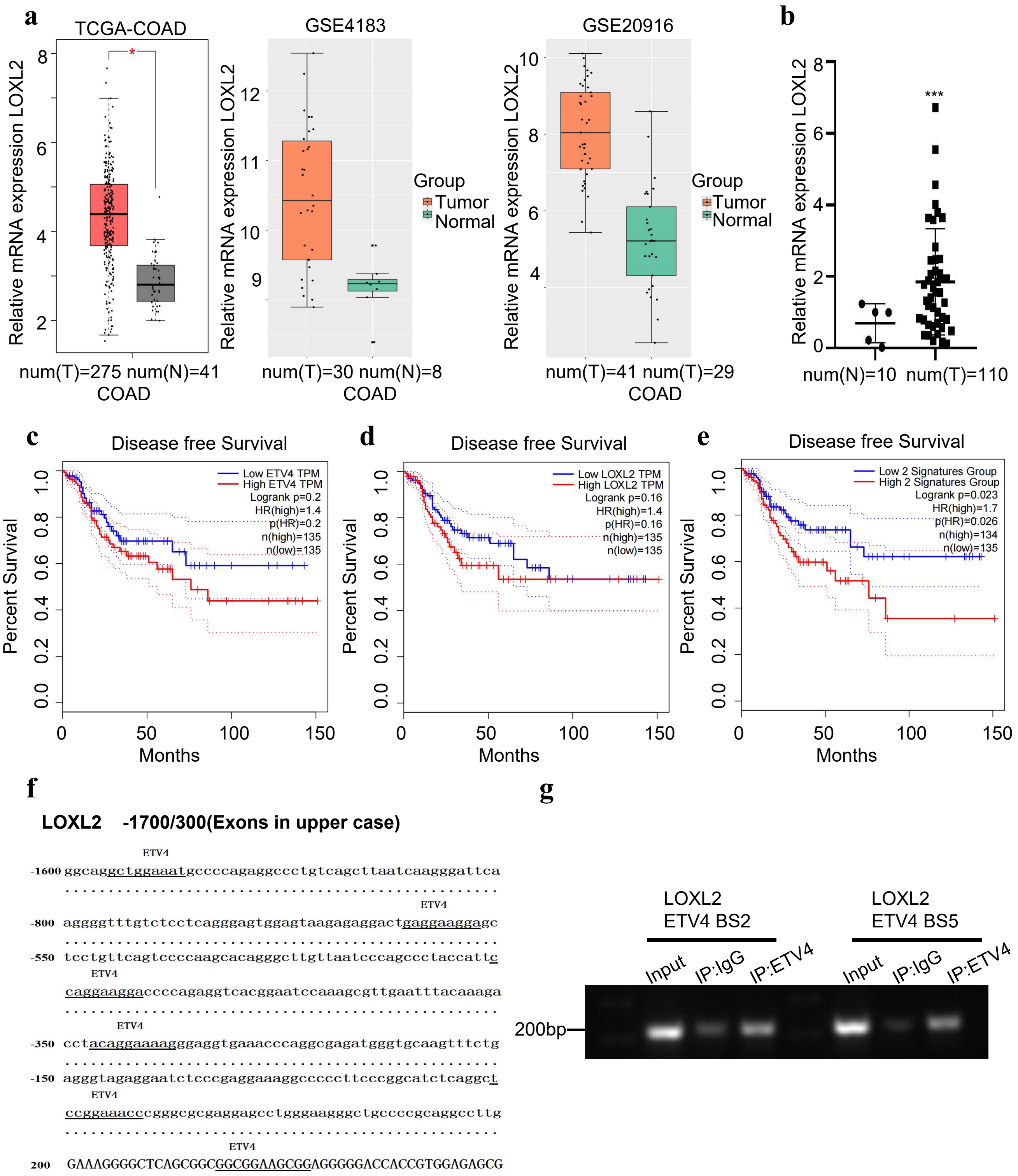
a
TCGA-COAD
GSE4183
GSE20916
Relative mRNA expression LOXL2
Group
Tumor
Normal
num(T)=275 num(N)=41
COAD
num(T)=30 num(N)=8
COAD
num(T)=41 num(T)=29
COAD
b
***
num(N)=10 num(T)=110
c
Disease free Survival
Low ETV4 TPM
High ETV4 TPM
Logrank p=0.2
HR(high)=1.4
p(HR)=0.2
n(high)=135
n(low)=135
Percent Survival
Months
d
Disease free Survival
Low LOXL2 TPM
High LOXL2 TPM
Logrank p=0.16
HR(high)=1.4
p(HR)=0.16
n(high)=135
n(low)=135
e
Disease free Survival
Low 2 Signatures Group
High 2 Signatures Group
Logrank p=0.023
HR(high)=1.7
p(HR)=0.026
n(high)=134
n(low)=135
f
LOXL2 -1700/300(Exons in upper case)
ETV4
-1600 ggcaggctggaaatgccccagaggccctgtcagcttaatcaagggattca
ETV4
-800 aggggtttgtctcctcagggagtggagtaagagaggactgaggaaggagc
-550 tcctgttcagtccccaagcacagggcttgttaatcccagccctaccattc
ETV4
caggaaggaccccagaggtcacggaatccaaagcgttgaatttacaaaga
ETV4
-350 cctacaggaaaaagggaggtgaaacccaggcgagatgggtgcaagtttctg
-150 agggtagaggaatctcccgaggaaaggccccccttcccggcatctcaggct
ETV4
ccggaaacccgggcgcgaggagcctgggaagggctgccccgcaggccttg
ETV4
200 GAAAGGGGCTCAGCGGCGGCGGAAGCGGAGGGGGACCACCGTGGAGAGCG
g
LOXL2
ETV4 BS2
LOXL2
ETV4 BS5
Input
IP:IgG
IP:ETV4
200bp

Figure S7

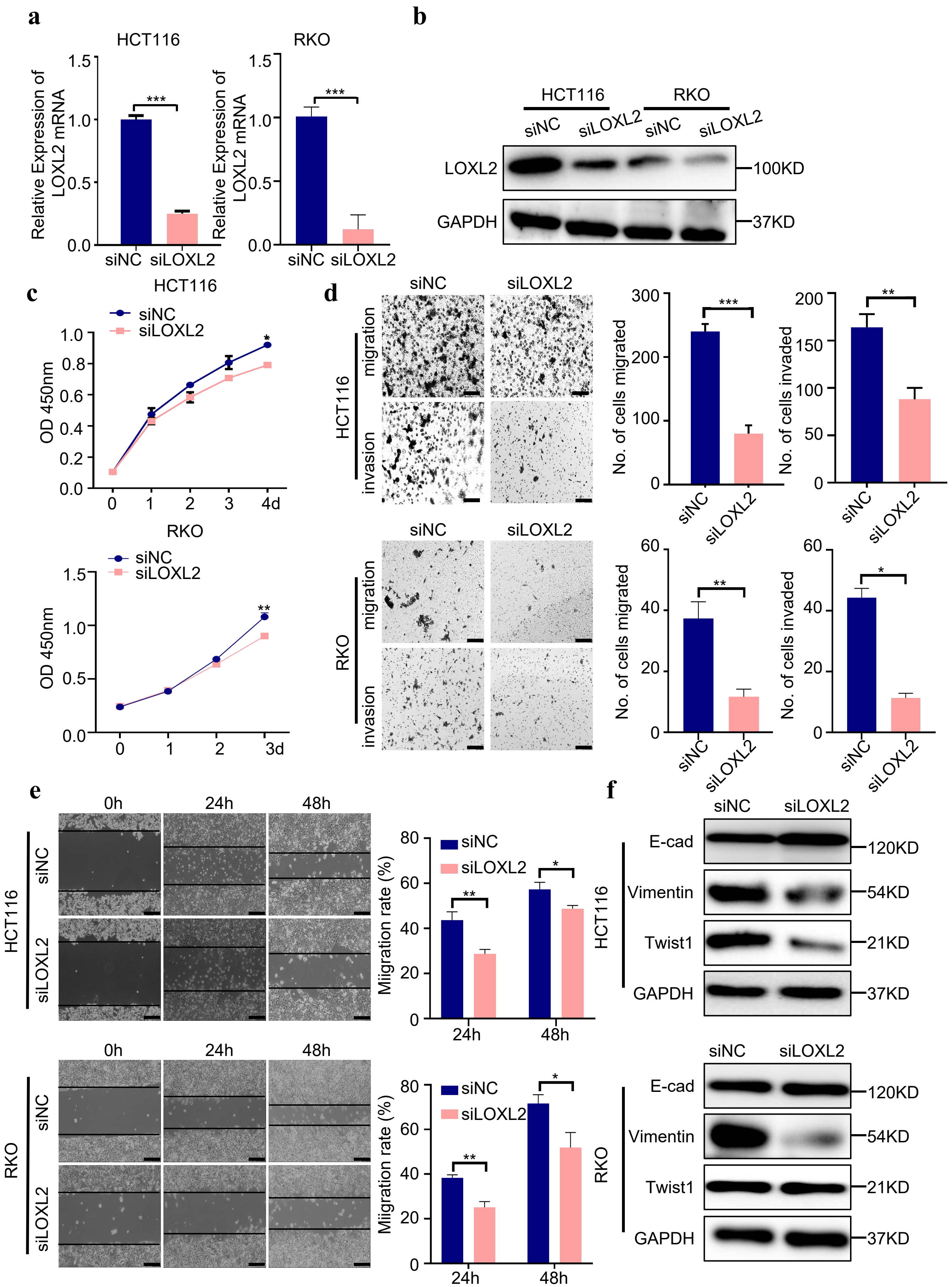
a
HCT116
RKO
Relative Expression of LOXL2 mRNA
1.5
1.0
0.5
0.0
***
siNC siLOXL2
b
HCT116
RKO
siNC siLOXL2 siNC siLOXL2
LOXL2
100KD
GAPDH
37KD
c
HCT116
siNC
siLOXL2
OD 450nm
1.0 0.8 0.6 0.4 0.2 0.0
0 1 2 3 4d
*
RKO
1.5 1.0 0.5 0.0
0 1 2 3d
**
d
siNC siLOXL2
HCT116
migration
invasion
RKO
No. of cells migrated
No. of cells invaded
300 200 100 0
200 150 100 50 0
60 40 20 0
***
**
*
e
0h 24h 48h
siNC
siLOXL2
HCT116
RKO
Migration rate (%)
80 60 40 20 0
24h 48h
f
siNC siLOXL2
E-cad
120KD
Vimentin
54KD
Twist1
21KD
GAPDH
37KD

Figure S8

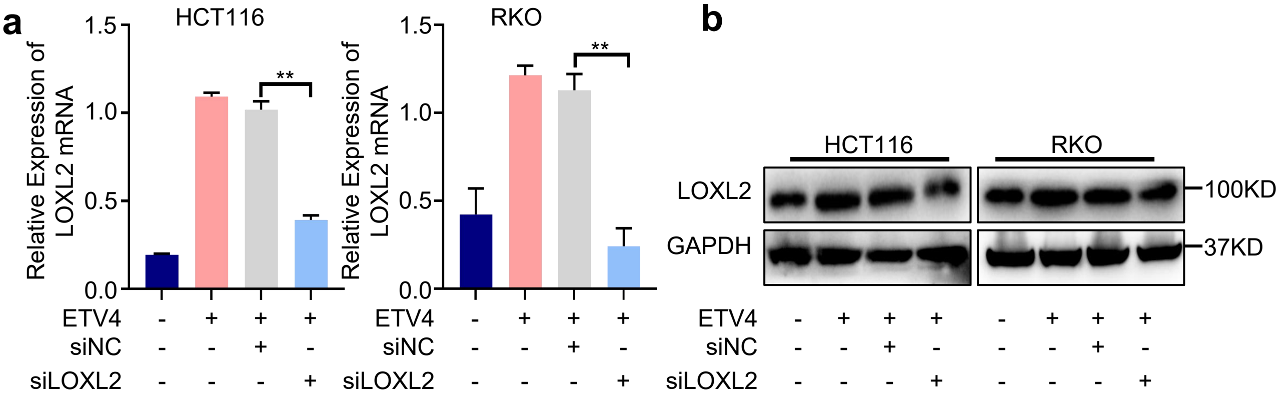

a
HCT116
RKO
Relative Expression of LOXL2 mRNA
1.5
1.0
0.5
0.0
**
ETV4 - + + +
siNC - - + -
siLOXL2 - - - +
b
HCT116
RKO
LOXL2
GAPDH
100KD
37KD
ETV4 - + + + - + + +
siNC - - + - - - + -
siLOXL2 - - - + - - - +

Figure S9

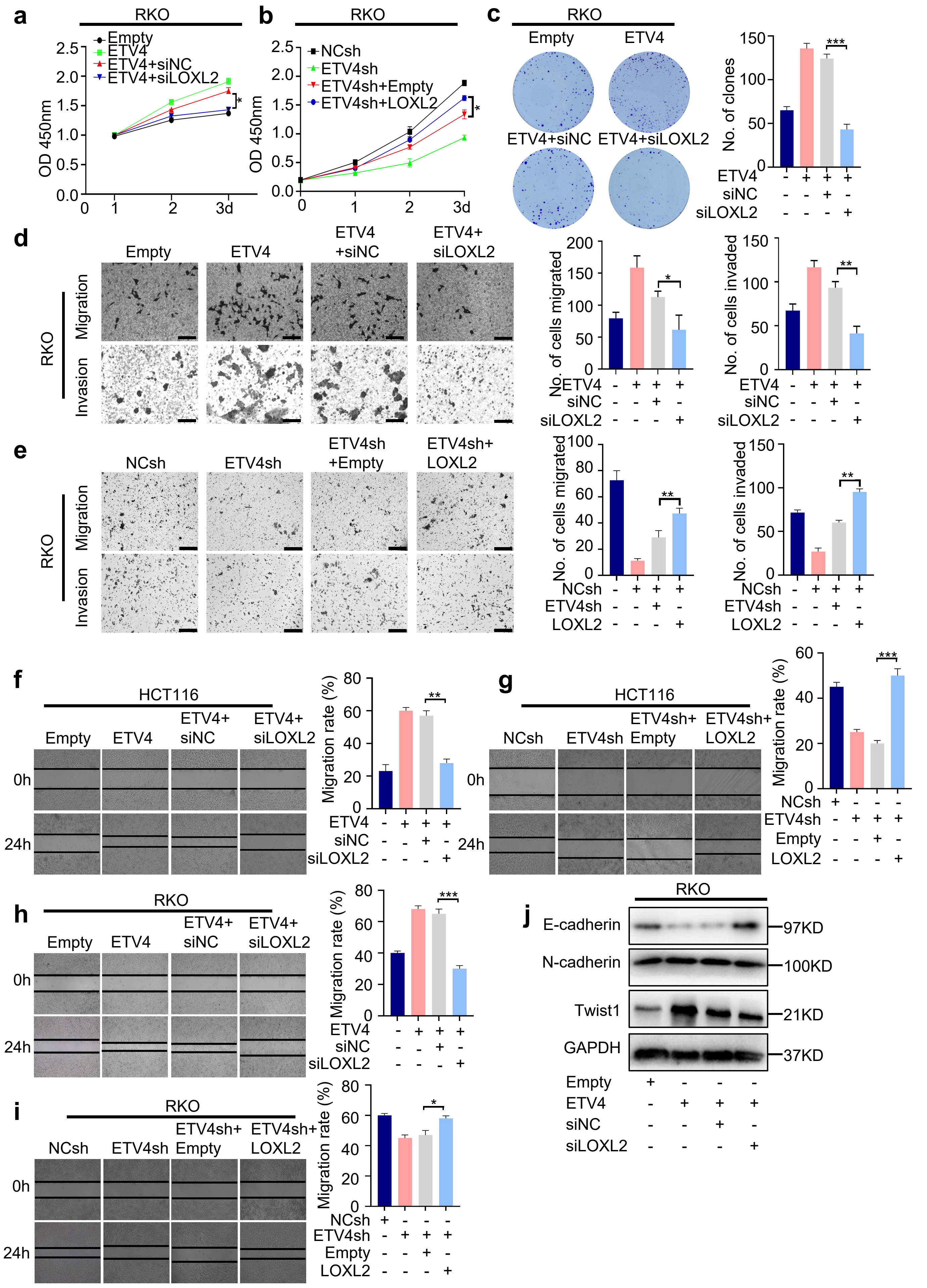
a
RKO
Empty
ETV4
ETV4+siNC
ETV4+siLOXL2
OD 450nm
b
RKO
NCsh
ETV4sh
ETV4sh+Empty
ETV4sh+LOXL2
OD 450nm
c
RKO
Empty
ETV4
ETV4+siNC
ETV4+siLOXL2
No. of clones
ETV4
siNC
siLOXL2
d
Empty
ETV4
ETV4 +siNC
ETV4+ siLOXL2
RKO
Migration
Invasion
No. of cells migrated
No. of cells invaded
e
NCsh
ETV4sh
ETV4sh +Empty
ETV4sh+ LOXL2
RKO
Migration
Invasion
No. of cells migrated
No. of cells invaded
LOXL2
f
HCT116
Migration rate (%)
0h
24h
g
HCT116
NCsh
ETV4sh
ETV4sh+ Empty
ETV4sh+ LOXL2
Migration rate (%)
h
RKO
Migration rate (%)
i
RKO
Migration rate (%)
j
RKO
E-cadherin
N-cadherin
Twist1
GAPDH
97KD
100KD
21KD
37KD
Empty
ETV4
siNC
siLOXL2

Figure S10

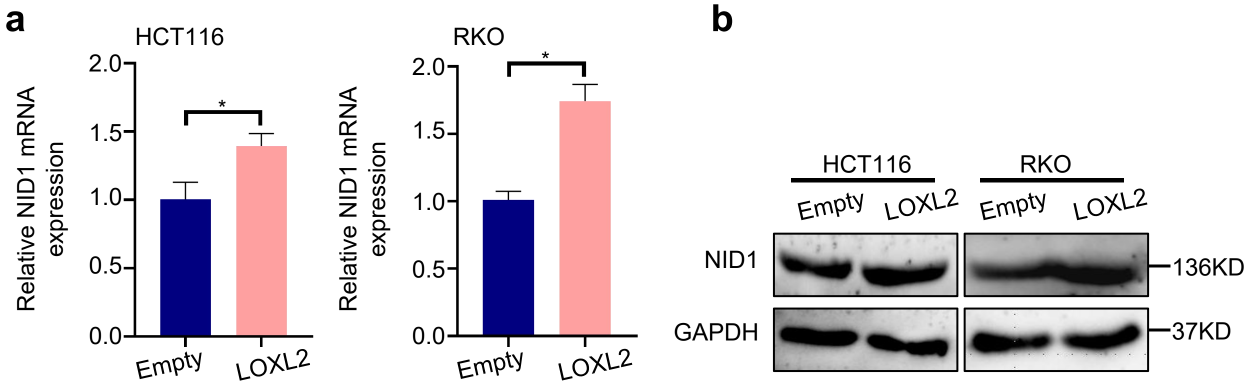

a
HCT116
Relative NID1 mRNA expression
2.0
1.5
1.0
0.5
0.0
*
Empty
LOXL2
RKO
Relative NID1 mRNA expression
2.0
1.5
1.0
0.5
0.0
*
Empty
LOXL2
b
HCT116
RKO
Empty
LOXL2
Empty
LOXL2
NID1
GAPDH
136KD
37KD

Figure S11

LOXL2 is part of cluster 47 **Fibroblasts - ECM organization** with confidence[i] 0.82

271 genes in cluster

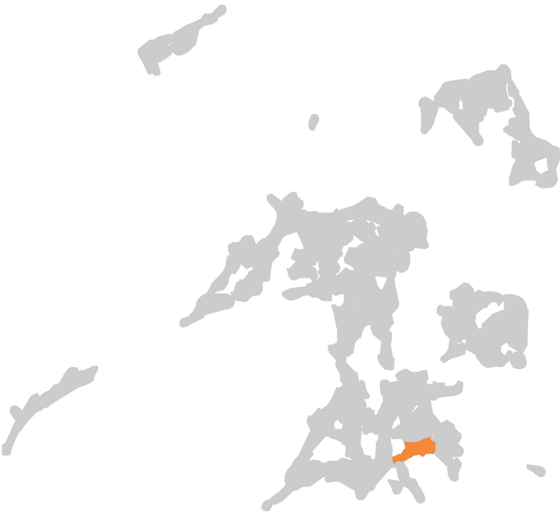

15 nearest neighbours based on tissue RNA expression

**Neighbour**[i]	**Description**[i]	**Correlation**[i]	**Cluster**[i]
CLMP	CXADR like membrane protein	0.9526	47
CTHRC1	Collagen triple helix repeat containing 1	0.9439	69
ITIH5	Inter-alpha-trypsin inhibitor heavy chain 5	0.9351	69
COL5A2	Collagen type V alpha 2 chain	0.9316	47
COL3A1	Collagen type III alpha 1 chain	0.9298	47
HAS2	Hyaluronan synthase 2	0.9281	47
GJC1	Gap junction protein gamma 1	0.9246	47
MMP2	Matrix metallopeptidase 2	0.9228	47
PCDH18	Protocadherin 18	0.9211	47
COL1A2	Collagen type I alpha 2 chain	0.9193	47
NID1	Nidogen 1	0.9193	47
LUM	Lumican	0.9035	47
MMRN1	Multimerin 1	0.9035	47
IL33	Interleukin 33	0.9035	47
FBN1	Fibrillin 1	0.9018	69

Figure S12

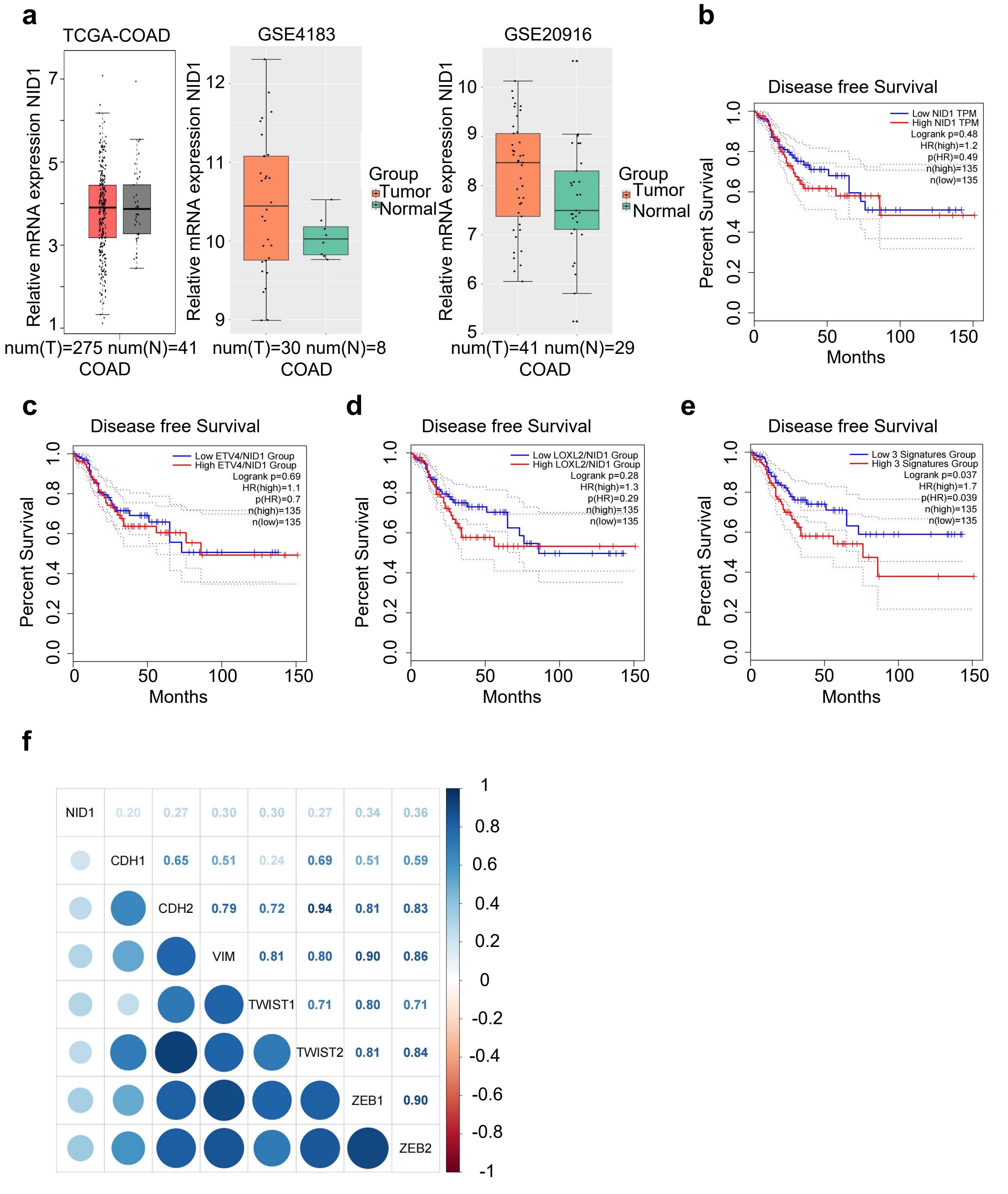
a
TCGA-COAD
GSE4183
GSE20916
Relative mRNA expression NID1
Group
Tumor
Normal
num(T)=275 num(N)=41
COAD
num(T)=30 num(N)=8
COAD
num(T)=41 num(N)=29
COAD
b
Disease free Survival
Low NID1 TPM
High NID1 TPM
Logrank p=0.48
HR(high)=1.2
p(HR)=0.49
n(high)=135
n(low)=135
Percent Survival
Months
c
Disease free Survival
Low ETV4/NID1 Group
High ETV4/NID1 Group
Logrank p=0.69
HR(high)=1.1
p(HR)=0.7
n(high)=135
n(low)=135
Percent Survival
Months
d
Disease free Survival
Low LOXL2/NID1 Group
High LOXL2/NID1 Group
Logrank p=0.28
HR(high)=1.3
p(HR)=0.29
n(high)=135
n(low)=135
Percent Survival
Months
e
Disease free Survival
Low 3 Signatures Group
High 3 Signatures Group
Logrank p=0.037
HR(high)=1.7
p(HR)=0.039
n(high)=135
n(low)=135
Percent Survival
Months
f
NID1 0.20 0.27 0.30 0.30 0.27 0.34 0.36
CDH1 0.65 0.51 0.24 0.69 0.51 0.59
CDH2 0.79 0.72 0.94 0.81 0.83
VIM 0.81 0.80 0.90 0.86
TWIST1 0.71 0.80 0.71
TWIST2 0.81 0.84
ZEB1 0.90
ZEB2

Figure S13

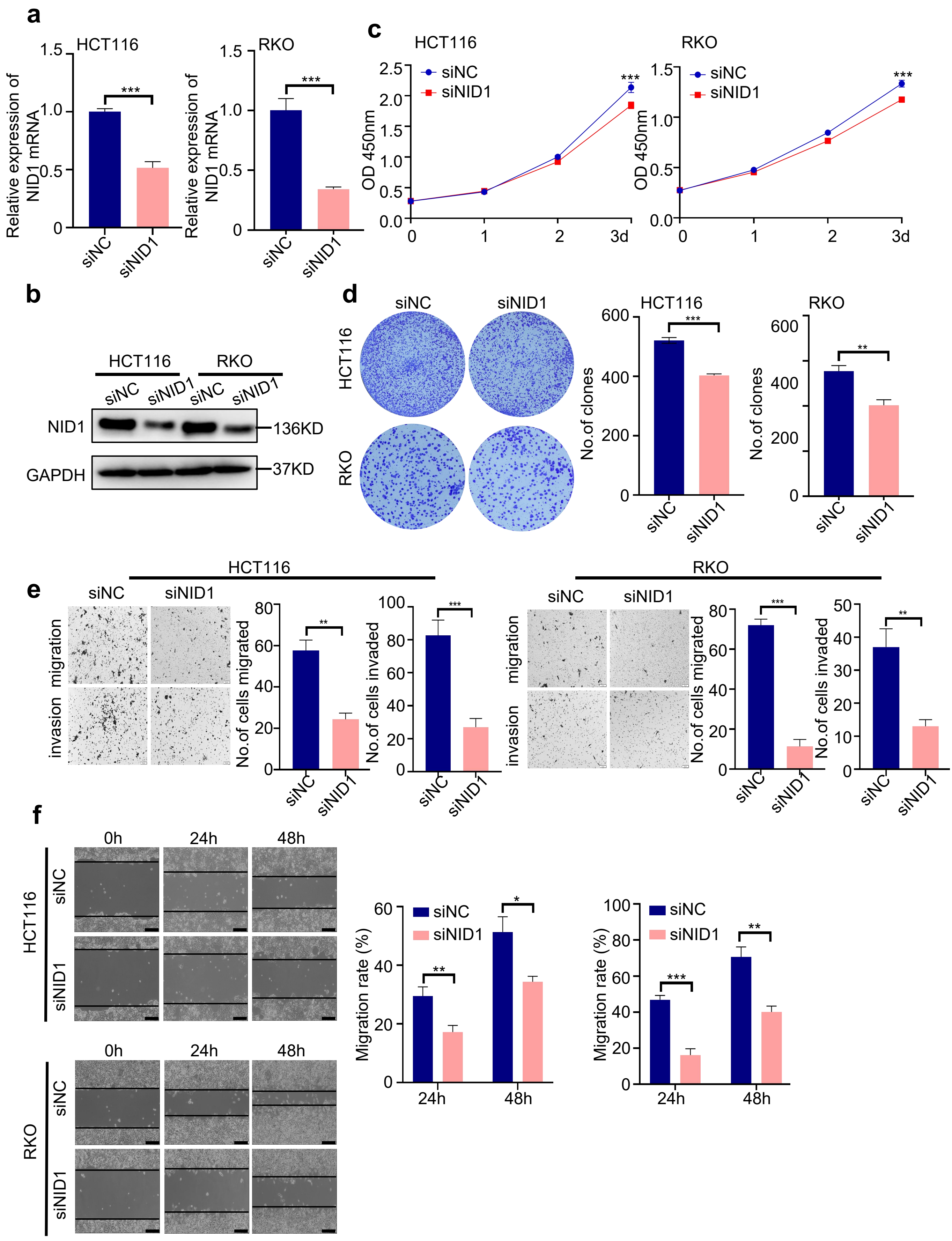
a
HCT116
RKO
Relative expression of NID1 mRNA
***
siNC
siNID1
c
HCT116
RKO
OD 450nm
siNC
siNID1
***
0
1
2
3d
b
HCT116
RKO
siNC
siNID1
NID1
136KD
GAPDH
37KD
d
siNC
siNID1
HCT116
RKO
No.of clones
***
**
e
HCT116
RKO
siNC
siNID1
migration
invasion
No.of cells migrated
No.of cells invaded
**
***
f
0h
24h
48h
HCT116
RKO
siNC
siNID1
Migration rate (%)
*
**
***
24h
48h

Figure S14

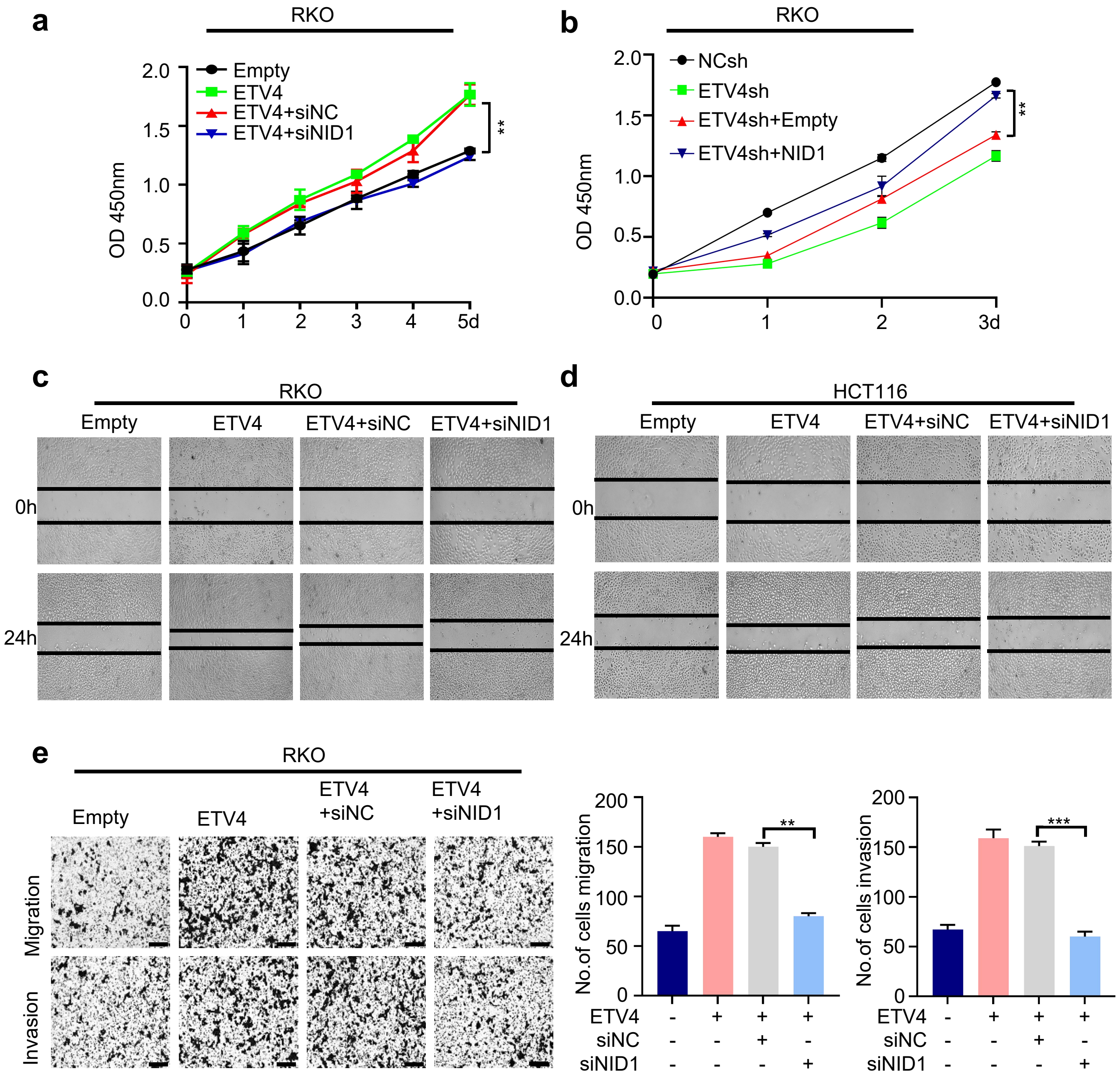
a
RKO
OD 450nm
Empty
ETV4
ETV4+siNC
ETV4+siNID1
**
5d
b
RKO
OD 450nm
NCsh
ETV4sh
ETV4sh+Empty
ETV4sh+NID1
**
3d
c
RKO
Empty
ETV4
ETV4+siNC
ETV4+siNID1
0h
24h
d
HCT116
Empty
ETV4
ETV4+siNC
ETV4+siNID1
0h
24h
e
RKO
Empty
ETV4
ETV4 +siNC
ETV4 +siNID1
Migration
Invasion
No.of cells migration
No.of cells invasion
ETV4
siNC
siNID1
**
***

Figure S15

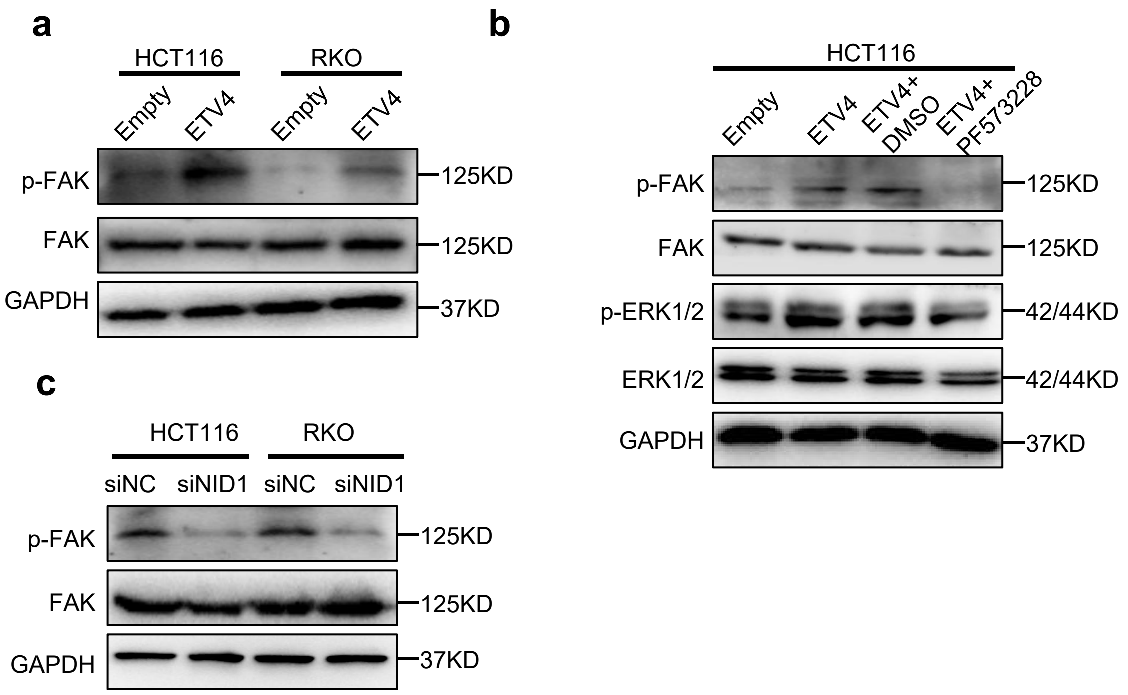
a
HCT116
RKO
Empty
ETV4
Empty
ETV4
p-FAK
125KD
FAK
125KD
GAPDH
37KD
b
HCT116
Empty
ETV4
ETV4+ DMSO
ETV4+ PF573228
p-FAK
125KD
FAK
125KD
p-ERK1/2
42/44KD
ERK1/2
42/44KD
GAPDH
37KD
c
HCT116
RKO
siNC
siNID1
siNC
siNID1
p-FAK
125KD
FAK
125KD
GAPDH
37KD

Figure S16

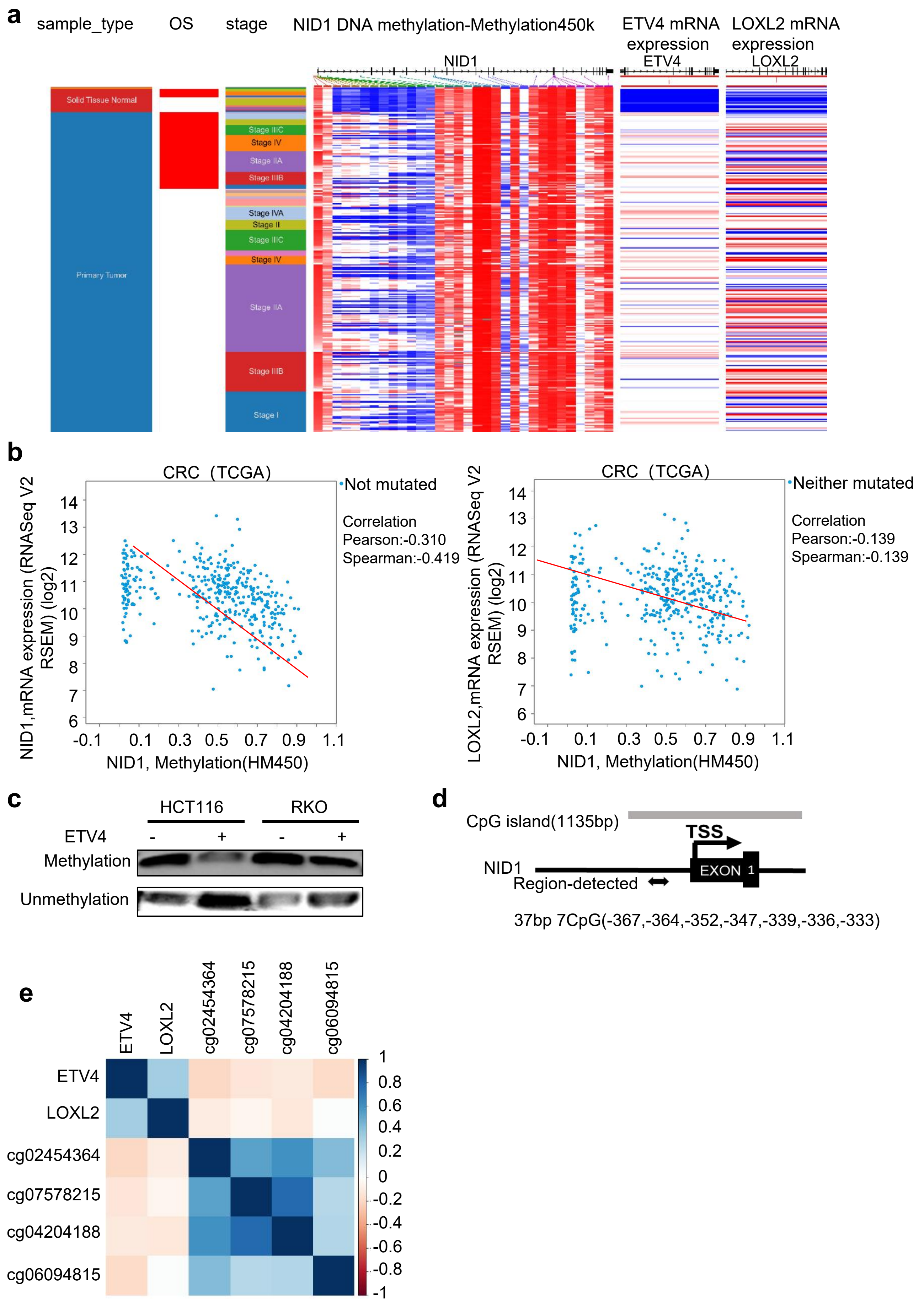
a
sample_type
OS
stage
NID1 DNA methylation-Methylation450k
ETV4 mRNA expression
LOXL2 mRNA expression
NID1
ETV4
LOXL2
Solid Tissue Normal
Primary Tumor
Stage IIIC
Stage IV
Stage IIA
Stage IIIB
Stage IVA
Stage II
Stage IIIC
Stage IV
Stage IIA
Stage IIIB
Stage I
b
CRC (TCGA)
Not mutated
Correlation
Pearson:-0.310
Spearman:-0.419
NID1,mRNA expression (RNASeq V2 RSEM) (log2)
NID1, Methylation(HM450)
CRC (TCGA)
Neither mutated
Correlation
Pearson:-0.139
Spearman:-0.139
LOXL2,mRNA expression (RNASeq V2 RSEM) (log2)
NID1, Methylation(HM450)
c
HCT116
RKO
ETV4
Methylation
Unmethylation
d
CpG island(1135bp)
TSS
NID1
EXON 1
Region-detected
37bp 7CpG(-367,-364,-352,-347,-339,-336,-333)
e
ETV4
LOXL2
cg02454364
cg07578215
cg04204188
cg06094815

Figure S17

a

NID1
-1300/300(Exons in upper case)

ETV4

-1250 caaggtcgttacttttagctgctgacaggaaacggggacgtcctgttgac
cgctgataggtctgacatcaaacatatccaactccagctgtcccagatcc

....

ETV4

-950 tcaaccgattattcaggataccacttaaatcgcttatctccctttgtcaa
gaactttcaatagtttcttctccgagtgacagagcgaattcaaattccta

....

ETV4

-450 cccgtcttcctaccagggtcaagcgaattggacccggacgcggccgagc
ggcaatggggtgggcgccgttcctgaatccggagcgtttccacgtcgccg

b

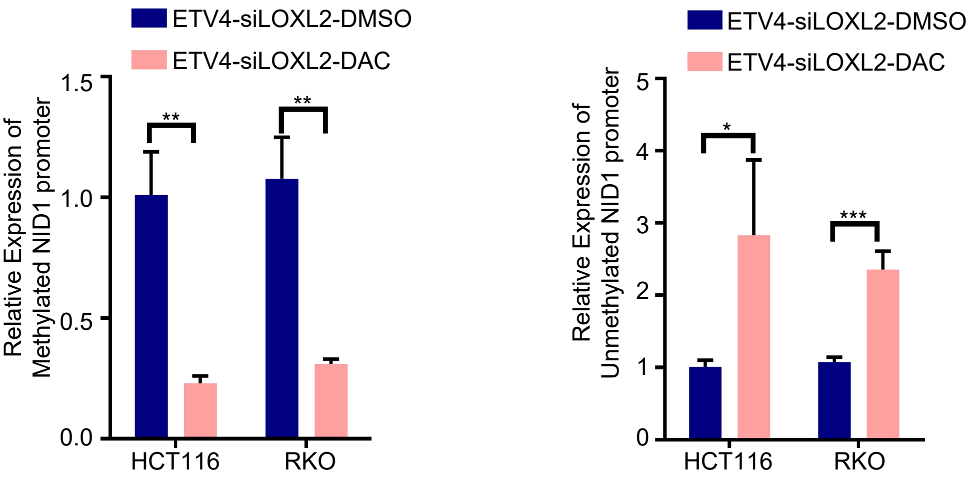

c

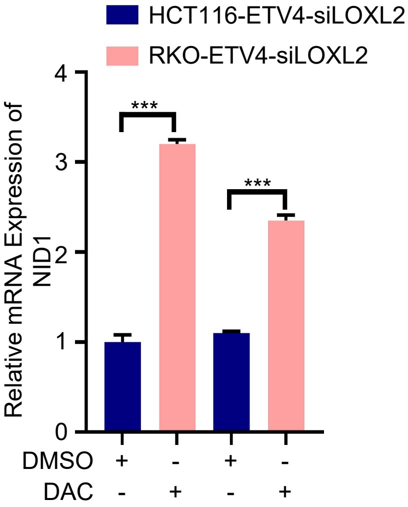

d

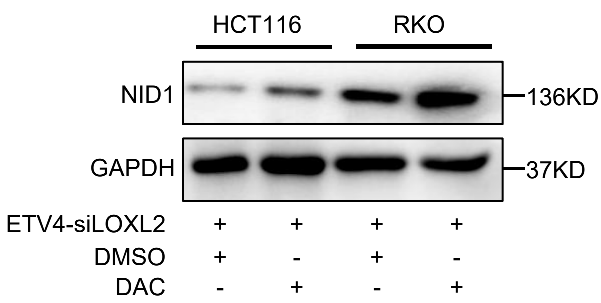

Supplement: Supplementary file 1 — Supplementary figures. [file ijbsv21p6674s1.pdf]
