# Supplementary material for: ETV4 interacts with LOXL2 to induce epigenetic activation of NID1 during colorectal cancer progression
Source: Int J Biol Sci. 2025 Oct 20;21(15):6674–96. doi: 10.7150/ijbs.116383 (PMC12631116; doi:10.7150/ijbs.116383)
Supplement: Supplementary file 2 — Supplementary tables. [file ijbsv21p6674s2.pdf]

Supplementary Tables

Supplementary Table S1. The clinical information of colorectal cancer tissue microarray.

#	Position	Sex	Age	Tissue	Pathological Diagnosis	Grade	Stage	TNM
1	A1	F	42	Colon	Adenocarcinoma	1	IIB	T4N0M0
2	A2	M	65	Colon	Mucinous adenocarcinoma	1	IIA	T3N0M0
3	A3	M	70	Colon	Adenocarcinoma	1	IIA	T3N0M0
4	A4	F	50	Colon	Adenocarcinoma	1	IIIC	T4N2M0
5	A5	F	53	Colon	Adenocarcinoma	1	IIB	T4N0M0
6	A6	M	34	Colon	Adenocarcinoma	1	IIB	T4N0M0
7	A7	M	72	Colon	Adenocarcinoma	1	I	T2N0M0
8	A8	M	51	Colon	Adenocarcinoma	1	IIIC	T4N2M0
9	A9	M	27	Colon	Adenocarcinoma	1	IIB	T4N0M0
10	A10	M	70	Colon	Adenocarcinoma	1	IIIB	T3N1M0
11	A11	F	60	Colon	Adenocarcinoma	-	IIA	T3N0M0
12	A12	F	53	Colon	Adenocarcinoma	1	IIA	T3N0M0
13	B1	M	71	Colon	Adenocarcinoma	1	IIA	T3N0M0
14	B2	M	60	Colon	Adenocarcinoma	1	IIA	T3N0M0
15	B3	F	30	Colon	Adenocarcinoma	1	IIIC	T3N2M0
16	B4	M	55	Colon	Adenocarcinoma	1	I	T2N0M0
17	B5	F	49	Colon	Adenocarcinoma	1	IIIB	T4N1M0
18	B6	M	75	Colon	Adenocarcinoma	1	IIB	T4N0M0
19	B7	M	44	Colon	Adenocarcinoma	-	IIB	T4N0M0
20	B8	M	82	Colon	Adenocarcinoma	1	IIB	T4N0M0
21	B9	M	86	Colon	Adenocarcinoma	1	IIIB	T4N1M0
22	B10	F	68	Colon	Adenocarcinoma	1	IIIB	T3N1M0
23	B11	F	65	Colon	Mucinous adenocarcinoma	1	IIA	T3N0M0
24	B12	M	61	Colon	Mucinous adenocarcinoma	1	IIA	T3N0M0
25	C1	F	59	Colon	Mucinous adenocarcinoma	1	IIIB	T3N1M0
26	C2	M	32	Colon	Mucinous adenocarcinoma	1	IIA	T3N1M0
27	C3	M	58	Colon	Mucinous adenocarcinoma	1	IV	T4N1M1
28	C4	M	55	Colon	Mucinous adenocarcinoma	2	IIIC	T3N2M0
29	C5	F	63	Colon	Adenocarcinoma	1	IIB	T4N0M0
30	C6	F	40	Colon	Mucinous adenocarcinoma	1	IIIB	T3N1M0
31	C7	M	71	Colon	Adenocarcinoma	2	IIIC	T4N2M0
32	C8	M	61	Colon	Adenocarcinoma	2	IIB	T4N0M0
33	C9	F	86	Colon	Adenocarcinoma	2	IIB	T4N0M0
34	C10	M	64	Colon	Mucinous adenocarcinoma	1	IIB	T4N0M0
35	C11	F	52	Colon	Adenocarcinoma	2	IIA	T3N0M0
36	C12	M	46	Colon	Adenocarcinoma	2	IIB	T4N0M0
37	D1	F	70	Colon	Adenocarcinoma	1	IIB	T4N0M0
38	D2	M	70	Colon	Adenocarcinoma	2	IIA	T3N0M0
39	D3	M	75	Colon	Adenocarcinoma	2	IIB	T4N0M0

40	D4	F	51	Colon	Adenocarcinoma (sparse)	-	IIB	T4N0M0
41	D5	M	60	Colon	Adenocarcinoma	2	IIB	T4N0M0
42	D6	F	41	Colon	Adenocarcinoma (sparse)	-	IIB	T4N0M0
43	D7	F	60	Colon	Adenocarcinoma	2	IIB	T4N0M0
44	D8	F	48	Colon	Adenocarcinoma	2	IIA	T3N0M0
45	D9	M	66	Colon	Mucinous adenocarcinoma	2	IIA	T3N0M0
46	D10	M	73	Colon	Adenocarcinoma	2	IIIB	T4N1M0
47	D11	M	75	Colon	Adenocarcinoma	2	IIA	T3N0M0
48	D12	M	37	Colon	Adenocarcinoma	-	IIIB	T4N1M0
49	E1	F	44	Colon	Adenocarcinoma	1	IIA	T3N0M0
50	E2	M	35	Colon	Adenocarcinoma	-	IIIB	T4N1M0
51	E3	F	53	Colon	Adenocarcinoma	1	IIA	T3N0M0
52	E4	M	70	Colon	Adenocarcinoma	1	IIA	T3N0M0
53	E5	F	40	Colon	Adenocarcinoma	1	IIB	T4N0M0
54	E6	M	53	Colon	Mucinous adenocarcinoma	3	IIA	T3N0M0
55	E7	M	28	Colon	Mucinous adenocarcinoma	3	IIB	T4N0M0
56	E8	F	71	Colon	Adenocarcinoma	2	IIA	T3N0M0
57	E9	M	58	Colon	Adenocarcinoma	2	IIB	T4N0M0
58	E10	M	74	Colon	Adenocarcinoma	2	IIA	T3N0M0
59	E11	M	69	Colon	Adenocarcinoma	2	IIIC	T4N2M0
60	E12	M	66	Colon	Adenocarcinoma	2	IIA	T3N0M0
61	F1	F	71	Colon	Mucinous adenocarcinoma	2	II	T3N0M0
62	F2	M	76	Colon	Mucinous adenocarcinoma	2	IIA	T3N0M0
63	F3	F	76	Colon	Adenocarcinoma	1	I	T2N0M0
64	F4	F	50	Colon	Mucinous adenocarcinoma	2	IIB	T4N0M0
65	F5	F	60	Colon	Mucinous adenocarcinoma	2	IIIB	T3N1M0
66	F6	F	49	Colon	Mucinous adenocarcinoma	3	IV	T4N1M1
67	F7	M	77	Colon	Adenocarcinoma with necrosis	2	IIIB	T3N1M0
68	F8	F	80	Colon	Adenocarcinoma	2	IIA	T3N0M0
69	F9	F	70	Colon	Adenocarcinoma	2	IIIC	T3N2M0
70	F10	M	30	Colon	Adenocarcinoma	2	IIIA	T2N1M0
71	F11	M	43	Colon	Adenocarcinoma	2	IIIB	T3N1M0
72	F12	M	50	Colon	Adenocarcinoma	2	IV	T2N1M1
73	G1	M	41	Colon	Adenocarcinoma	2	IIIC	T4N2M0
74	G2	M	66	Colon	Adenocarcinoma with necrosis	-	IIA	T3N0M0
75	G3	M	79	Colon	Adenocarcinoma	1	IIA	T3N0M0
76	G4	M	49	Colon	Adenocarcinoma	1	IIB	T4N0M0
77	G5	F	56	Colon	Adenocarcinoma	-	IIA	T3N0M0
78	G6	F	73	Colon	Adenocarcinoma	2	IIIC	T4N2M0
79	G7	M	77	Colon	Adenocarcinoma	2	IIA	T3N0M0
80	G8	M	50	Colon	Adenocarcinoma	1	I	T2N0M0
81	G9	M	48	Colon	Adenocarcinoma	2	IIA	T3N0M0
82	G10	M	44	Colon	Adenocarcinoma	2	IIB	T4N0M0
83	G11	M	31	Colon	Adenocarcinoma	2	IIB	T4N0M0

84	G12	M	82	Colon	Adenocarcinoma	2	IIA	T3N0M0
85	H1	M	71	Colon	Adenocarcinoma	2	IIIC	T4N2M0
86	H2	M	62	Colon	Adenocarcinoma	2	IIA	T3N0M0
87	H3	M	47	Colon	Adenocarcinoma	2	IIB	T4N0M0
88	H4	M	55	Colon	Adenocarcinoma	2	IIIB	T3N1M0
89	H5	M	62	Colon	Adenocarcinoma	2	IV	T4N0M1
90	H6	M	38	Colon	Adenocarcinoma	2	IIIB	T4N1M0
91	H7	M	50	Colon	Adenocarcinoma	2	II	T4N0M0
92	H8	M	70	Colon	Mucinous adenocarcinoma	2	IIB	T4N0M0
93	H9	F	22	Colon	Adenocarcinoma	3	IV	T4N2M1
94	H10	M	58	Colon	Adenocarcinoma	3	IIIB	T3N1M0
95	H11	M	33	Colon	Adenocarcinoma	3	IIA	T3N0M0
96	H12	M	72	Colon	Adenocarcinoma	3	IIB	T4N0M0
97	I1	M	68	Colon	Adenocarcinoma	3	IV	T4N0M1
98	I2	M	54	Colon	Mucinous adenocarcinoma	3	IV	T3N1M1
99	I3	M	24	Colon	Mucinous adenocarcinoma	3	IIIC	T3N2M0
100	I4	M	56	Colon	Adenocarcinoma	3	IIA	T3N0M0
101	I5	M	45	Colon	Mucinous adenocarcinoma	3	IIA	T3N0M0
102	I6	M	68	Colon	Adenocarcinoma	3	III	T3N1M0
103	I7	M	36	Colon	Adenocarcinoma	3	IIB	T4N0M0
104	I8	F	74	Colon	Adenocarcinoma with necrosis	3	IIIB	T4N1M0
105	I9	M	60	Colon	Adenocarcinoma with necrosis	3	IIA	T3N0M0
106	I10	F	53	Colon	Adenocarcinoma	3	IIB	T4N0M0
107	I11	M	49	Colon	Mucinous adenocarcinoma	3	IIIB	T4N1M0
108	I12	M	71	Colon	Adenocarcinoma	2	IIIB	T4N1M0
109	J1	M	75	Colon	Adenocarcinoma	3	IIB	T4N0M0
110	J2	F	31	Colon	Adenocarcinoma (sparse)	-	IV	T4N1M1
111	J3	M	35	Colon	Colon tissue	-	-	-
112	J4	M	25	Colon	Colon tissue	-	-	-
113	J5	M	35	Colon	Colon tissue	-	-	-
114	J6	M	30	Colon	Colon tissue	-	-	-
115	J7	M	30	Colon	Colon tissue	-	-	-
116	J8	M	40	Colon	Colon tissue	-	-	-
117	J9	M	28	Colon	Colon tissue	-	-	-
118	J10	M	45	Colon	Colon tissue	-	-	-
119	J11	M	33	Colon	Colon tissue	-	-	-
120	J12	M	30	Colon	Colon tissue	-	-	-

Supplementary Table S2. Antibodies used in the present study.

Protein name	Manufacture (cat. number)	Manufacture (cat. number)	Website Link
GAPDH	Xianzhi Bio(AB-P-R 001)	IB(1:5000)	http://www.goodhere.com/showproduct.asp?id=320&classid=34&nid=2
GAPDH	Proteintech（10494-1-AP）	IB(1:5000)	https://www.ptgcn.com/products/GAPDH-Antibody-10494-1-AP.htm
ETV4	Proteintech（10684-1-AP）	IB(1:2000)<br>IHC-P(1:200)	https://www.ptglab.com/products/ETV4-Antibody-10684-1-AP.htm
ETV4	Abcam(ab70425)	IF(1:200)	http://www.abcam.cn/pea3-antibody-1a2g3-ab70425.html
ETV4	Sigma (SAB1403795)	IB(1:1000)	https://www.sigmaaldrich.cn/CN/zh/product/sigma/sab1403795?context=product
ETV4	Santa Cruz(sc-113)	IB(1:500)<br>IF(1:200)	https://www.scbt.com/p/pea3-antibody-16?requestFrom=search
LOXL2	Abcam(ab96233)	IB(1:1000)<br>IF(1:200)<br>ChIP(1:500)	http://www.abcam.cn/loxl2-antibody-ab96233.html
LOXL2	CST(99680)	IB(1:1000)	https://www.cellsignal.cn/products/primary-antibodies/loxl2-e3p7y-rabbit-mab/99680
E-cad	Origene (TA800692)	IB(1:2000)<br>IF(1：200)	http://www.origene.com/antibody/CDH1-TA800692.aspx
E-cad	Affinity(AF0131)	IB(1:1000)	https://www.affbiotech.cn/goods-56-AF0131-E_cadherin_Antibody.html
N-cad	Origene (TA503933)	IB(1:2000)<br>IF(1:200)	http://www.origene.com/antibody/CDH2-TA503933.aspx
N-cad	Affinity(AF5239)	IB(1:1000)	https://www.affbiotech.cn/goods-4546-AF5239-N_Cadherin_Antibody.html
Twist	Abcam(ab175430)	IB(1:1000)	http://www.abcam.cn/twist-antibody-10e4e6-ab175430.html
Vimentin	CST（5741s）	IB(1:1000)<br>IF(1:200)	http://www.cst-c.com.cn/products/5741.html
Vimentin	Proteintech（60330-1-Ig）	IB(1:2000)	https://www.ptgcn.com/products/Vimentin-Antibody-60330-1-Ig.htm
PCNA	Ruiying Biological (RLM3031)	IHC-P(1:200)	http://rlgene.com/showproduct.asp?/1_172
ERK1/2	CST (1240)	IB(1:2000)	http://www.cst-c.com.cn/products/1240.html
P-ERK1/2	CST(4695S)	IB(1:2000)	http://www.cst-c.com.cn/products/4695.html
NID1	Proteintech（13766-1-AP）	IB (1:500)	https://www.ptgcn.com/products/NID1-Antibody-13766-1-AP.htm
Flag	Sigma Aldrich	IP（1:50）	https://www.sigmaaldrich.com/catalog/product/

	(F1804-200UG)	IB(1:1000)	sigma/f1804?lang=zh&region=CN
DNMT1	GeneTex (GTX116011)	IB(1:1000)	https://www.genetex.cn/Product/Detail/DNMT1-antibody-N1-N-term/GTX116011
TET1	GeneTex (GTX627420)	IB(1:1000)	https://www.genetex.cn/Product/Detail/TET1-antibody-GT1462/GTX627420
P300 (D2X6N)	CST(#54062S)	ChIP(1:500)	https://www.cellsignal.cn/products/primary-antibodies/p300-d2x6n-rabbit-mab/54062?site-search-type=Products&N=4294956287&Ntt=54062s&fromPage=plp&_requestid=1169368
Anti-Mouse seccondary antibody	Abgent(ASS1007)	IB(1:5000)	http://www.abgent.com/products/ASS1007-Goat-Anti-Mouse-IgGHL-Human-ads-HRP-Secondary-Antibody
Anti-Rabbit secondary antibody	Abgent (ASS1009)	IB (1:5000)	http://www.abgent.com/products/ASS1009-Goat-Anti-Rabbit-IgGHL-MouseHuman-ads-HRP-Secondary-Antibody
Anti-Mouse seccondary antibody	Thermo(A-11031)	IF(1:1000)	https://www.thermofisher.com/antibody/product/Goat-anti-Mouse-IgG-H-L-Secondary-Antibody-Polyclonal/A-11031
Anti-Rabbit seccondary antibody	Thermo(A-11034)	IF(1:1000)	https://www.thermofisher.com/antibody/product/Goat-anti-Rabbit-IgG-H-L-Secondary-Antibody-Polyclonal/A-11034
PEA3	Santa Cruz (SC-126X)	ChIP(1:500)	http://www.scbt.com/datasheet-126-p53-do-1-antibody.html
Normal mouse IgG	Millipore (12-271)	ChIP(1:500)	http://www.merckmillipore.com/CN/zh/product/Normal-Mouse-IgG,MM_NF-12-371
HA	CST (3724S)	IB(1:1000)	https://www.cellsignal.com/products/primary-antibodies/ha-tag-c29f4-rabbit-mab/3724?site-search-type=Products&N=4294956287&Ntt=c29f4&fromPage=plp
Flag	CST (14793S)	IB(1:1000)	https://www.cellsignal.com/products/primary-antibodies/dykddddk-tag-d6w5b-rabbit-mab-binds-to-same-epitope-as-sigma-s-anti-flag-m2-antibody/14793?site-search-type=Products&N=4294956287&Ntt=14793s&fromPage=plp&_requestid=2607052
FAK	CST (3285P)	IB(1:1000)	https://www.cellsignal.cn/products/primary-antibodies/fak-antibody/3285?site-search-type=Products&N=4294956287&Ntt=3285p&fromPage=plp&_requestid=2477034
P-FAK	CST (8556P)	IB(1:1000)	https://www.cellsignal.cn/products/primary-antibodies/phospho-fak-tyr397-d20b1-rabbit-mab/8556?site-search-type=Products&N=4294956287&Ntt=8556p&fromPage=plp&_requestid=2477240
P-FAK	Invitrogen(700154)	IB(1:1000)	https://www.thermofisher.cn/cn/zh/antibody/product/Phospho-FAK-Tyr861-Antibody-clone-26H16L4-Recombinant-Monoclonal/700154

Supplementary Table S3. The clinical pathological information of Colorectal Cancer cDNA Array.

#	NO.	Gender	Stage
1	C1	Male	0
2	C2	Male	0
3	C3	Male	0
4	C4	Female	0
5	C5	Female	0
6	C6	Male	I
7	C7	Female	I
8	C8	Female	I
9	C9	Female	I
10	C10	Female	I
11	C11	Female	I
12	C12	Male	II
13	D1	Male	IIA
14	D2	Female	IIA
15	D3	Female	IIA
16	D4	Female	IIA
17	D5	Male	IIA
18	D6	Female	IIA
19	D7	Female	IIA
20	D8	Male	IIA
21	D9	Male	IIA
22	D10	Male	IIA
23	D11	Male	IIA
24	D12	Male	IIA
25	E1	Male	IIA
26	E2	Male	IIA
27	E3	Female	IIB
28	E4	Male	IIB
29	E5	Male	IIB
30	E6	Male	III
31	E7	Male	III
32	E8	Female	III
33	E9	Female	III
34	E10	Female	III
35	E11	Female	IIIA
36	E12	Female	IIIA
37	F1	Male	IIIA
38	F2	Female	IIIB
39	F3	Male	IIIB

40	F4	Female	IIIB
41	F5	Male	IIIC
42	F6	Male	IIIC
43	F7	Male	IIIC
44	F8	Male	IV
45	F9	Male	IV
46	F10	Female	IV

Supplementary Table S4. List of primer sequences used for RT-qPCR analyses.

Gene name	Primer name and sequences
GAPDH	F833: 5'-ACCTGACCTGCCGTCTAGAA-3'
	R1060: 5'-TCCACCACCCTGTTGCTGTA-3'
ETV4	F664: 5'-AGCAGTGCCTTTACTCCAGT-3'
	R899: 5'-CCCTCCCTGAGATGTGAAGG-3'
	F1221: 5'-GATGATGTCTGCGTTGTCCC-3'
	R1385: 5'-CCAGGCAATGAAATGGGCAT-3'
	F1199: 5'-GAAACCTCTGCGACCATTCC -3'
	R1351: 5'- AGCAAGGCCACCAGAAATTG -3'
LOXL2	F1274: 5'-CCTCAAGATTCCGGAAAGCG-3'
	R1522: 5'-ACTGGATCTCGTTGAGGTGG-3'
E-cad	F764: 5'-TGAAGGTGACAGAGCCTCTGGAT-3'
	R915: 5'-TGGGTGAATTCGGGCTTGTT-3'
N-cad	F1685: 5'-ATCAACCCCATACACCAGCC-3'
	R1790: 5'-ACTAACCCGTCGTTGCTGTT-3'
Vimentin	F1369: 5'-CCAAACTTTTCCTCCCTGAACC-3'
	R1510: 5'-GTGATGCTGAGAAGTTTCGTTGA-3'
Snail	F32: 5’-GGTTCTTCTGCGCTACTGCT -3’
	R138: 5’-GTTAGGCTTCCGATTGGGGT -3’
Twist1	F1315: 5’-TCTCAAGAGGTCGTGCCAAT -3’
	R1457: 5’-ATGGTTTTGCAGGCCAGTTT-3’
Twist2	F1246: 5'-TCTCCGTGATTGCTTGGCTA-3'
	R1374: 5'-AGCAGGATACACAGCCACAC-3'
Zeb1	F209: 5’-GAGGATGACCTGCCAACAGA -3’
	R314: 5’-CTTGCCCTTCCTTTCCTGTG -3’
Zeb2	F803: 5’-AACACCCCTGGCACAACAA -3’
	R912: 5’-AATTGCGGTCTGGATCGTGG -3’
ARNTL2	F1174: 5'-TACTTGAGAAGCTGGCCTCC-3'
	R1357: 5'-TTCCATTCACTGCAAACCGG-3'
PER3	F795: 5'-GGTATTCTACGCGCACACTG-3'
	R1024: 5'-TGAGACAGCAAGGTTCCGAT-3'
THRB	F1345: 5'-TTTGACCTGGGCATGTCTCT-3'
	R1584: 5'-GGCTCCTATCATCCGCAGAT-3'
ADRB2	F964: 5'- CCTTACCTCCTTCTTGCCCA-3'
	R1142: 5'-CCCTGGAGTAGACGAAGACC-3'
E2F2	F809: 5'-ACTCGGTATGACACTTCGCT-3'
	R1036: 5'-TCTGGTGGGGTCTTCAAACA-3'
NRP1	F1501: 5'-ACAGGTAGACTTGGGCCTTC-3'
	R1694: 5'-CAACATCTGTGGGGTTGGTG-3'
WISP2	F1025: 5'-GTCGCAGTCCACAAAACAGT-3'
	R1184: 5'-GGTGGACCCAAGCTAAAGTG-3'

COL11A1	F1275: 5'-GCCTGGTATGCTTGTCGAAG-3'
	R1508: 5'-TCCTGAGCAGAGATGGTTGG-3'
DLC1	F606: 5'-AACTCCGTCATCAGCGTTTG-3'
	R766: 5'-TCCATCCGTTTCAGCAGACT-3'
FGF2	F624: 5'-AGGAGTGTGTGCTAACCGTT-3'
	R790: 5'-CAGTTCGTTTCAGTGCCACA-3'
KRAS	F17: 5'-TTGTGGTAGTTGGAGCTGGT-3'
	R182:5'- TGACCTGCTGTGTCGAGAAT-3'
NID1	F388:5'-TTATCCCCCTCCATCACTCA-3'
	R539: 5'-CTCTTGCCTTTCTGGTCTGG-3'
NRG1	F453: 5'-CTGTGTGAATGGAGGGGAGT-3'
	R692: 5'-TAGGCCACCACACACATGAT-3'
PDGFRB	F2615:5'- CGGAGAGCATCTTCAACAGC-3'
	R2848:5'- GGGGCCGAATCTCAAACTTC-3'

PCR primers used for MSP

Gene name	Primer name and sequences
NID1_MF	5'- GAGGGTTTCGTTTCGTTTAGC-3'
NID1_MR	5'- AACGCCGTTCGCTAAAAATCG-3'
NID1_UF	5'- GAGGGTTTTGTTTTGTTTAGT-3'
NID1_UR	5'- AAAAACACCATTCACTAAAAATCA-3'
GAPDH-UF	5'- GGTAAGGTTATTTTTGAGTTGAATG-3'
GAPDH-UR	5'- CATATTTAACAAATTTTTCTAAACAAC-3'

Primers used for ChIP analysis

Primer name	Primer sequence
LOXL2-ChIP-EBS2	F:5'-TGGGCTGGAGGTGAGATT-3'
	R:5'-CAGCGAGCTGCAAAACAA-3'
LOXL2-ChIP-EBS5	F:5'-GGAGGAAAGGGTAGAGGAA-3'
	R:5'-GAGGAGGATGGCAAACCC-3'
NID1-ChIP-EBS1	F:5'-GCTCTGTCCCTTGGCTGATT-3'
	R:5'-ATTAGGGTTGGGTCGTGGAGA-3'
NID1-ChIP-EBS2	F:5'-TTCTCCTCTTCAATGCCAATCTT-3'
	R:5'-TGGTATCCTGAATAATCGGTTGA-3'
NID1-ChIP-EBS3	F:5'-TAGGTTCAAGGTCGTTACTTTTAGC-3'
	R:5'-GATTGGCATTGAAGAGGAGAAGA-3'

Supplementary Table S5. **List of siRNA sequences.**

Name	Sequences
Negative control siRNA	Sense:5'-UUCUCCGAACGUGUCACGUUU-3'
	Antisense:5'-AAACGUGACACGUUCGGAGAA-3'
ETV4 siRNA	Sense:5'-AAGGGUGGCUACUCUUACUTT-3'
	Antisense:5'-AGUAAGAGUAGCCACCCUUTT-3'
LOXL2 siRNA	Sense:5'-GGAGGACACAGAAUGUGAATT-3'
	Antisense:5'-UUCACAUUCUGUGUCCUCCTT-3'
NID1 siRNA	Sense:5'-CCUCCACUCUUACGUAGUAAUTT-3'
	Antisense:5'-AUUACUACGUAAGAGUGGAGGTT-3'

Supplementary Table S6. Primers for recombinant plasmids.

Primers for luciferase reporter constructions

Constructs	Methods	Primer and/or Enzymes used
LOXL2-P1773	PCR based cloning	F849:ATTTCTCTATCGATA*GGTACC*GTGAGATGTGGTTGGCAGTT<br>*KpnI*<br>R2622:GCTTACTTAGATCGC*AGATCT*CACCAAGCGTAGGTAGCC<br>*BglII*
NID1-P2355	PCR based cloning	F891:ATTTCTCTATCGATA*GGTACC*GGATGGACCAATAGGAGTTT<br>*KpnI*<br>R3244:GCTTACTTAGATCGC*AGATCT*CGATGTCGGATCTGTCGTAG<br>*BglII*

Primers for mutant constructions

Name	Sequences
LOXL2-P1773-EBS2m	Sense:5'-GTCTTCCTTCGCCGCCTTTCAGGTGACA-3'
	Antisense:5'-GTCCTCTCTTACTCCACTCCCTGAGGAGACAA-3'
LOXL2-P1773-EBS5m	Sense:5'-GAATTCCCAACGGGCGCGAGGAGCC-3'
	Antisense:5'-GCCTGAGATGCCGGGAAGGGGGCCTTTC-3'
NID1-P2355-EBS1m	Sense:5'-CACTTCCCATGGGACGTCCTGTTGACCGCTGA-3'
	Antisense:5'-CAGCAGCTAAAAGTAACGACCTTGAACCTAGTT-3'
NID1-P2355-EBS1m	Sense:5'-GACTTCGCAAACTTAAATCGCTTATCTCCCTTTGTCAAGA-3'
	Antisense:5'-ATAATCGGTTGATCGTTACTTTGGCACTAGGGTG-3'
NID1-P2355-EBS1m	Sense:5'-AAATTCATATGCCGAGCGGCAATGGGG-3'
	Antisense:5'-GTCCAATTCGCTTGACCCTGGTAGGAAGACGGGGTGGG-3'

Primers for truncated constructions

Name	Sequences
pcDNA3.0-Flag-ETV4(1-339)	Sense:5'-GACCCAGCTTTCTTGTACAAAGTG-3'
	Antisense:5'-ACCCCGGCGCTGGTAG-3'
pcDNA3.0-Flag-ETV4(278-484)	Sense:5'-ATGTACCTCCACACAGAGGGCTTC-3'
	Antisense:5'-CTTATCGTCGTCGTCCTTGTAATCC-3'
pcDNA3.0-Flag-ETV4(340-484)	Sense:5'-GCCCTGCAGCTGTGGCAATT-3'
	Antisense:5'-CTTATCGTCGTCGTCCTTGTAATCCA-3'
GST-ETV4(1-339)	Sense:5'-CG*GGATCC*ATGGAACGTCGCATGAAAGCAGGCT-3' *BamH I*
	Antisense:5'-CCG*CTCGAG*ACCGCGGCGCTGATACGGCGGA-3' *XhoI*

Supplementary Table S7. The transcription factors of common differentially expressed genes (DEGs) among three CRC datasets

Gene	GSE4183_ logFC	GSE20916_ logFC	TCGA-COAD_ logFC	Species	ensembl_gene_id
FOXQ1	2.416587657	6.988616745	5.402373972	Homo_sapiens	ENSG00000164379
ETV4	1.783844399	2.571062609	4.72539579	Homo_sapiens	ENSG00000175832
MSX2	1.385191547	3.07503765	3.778447214	Homo_sapiens	ENSG00000120149
ZIC2	1.315136922	1.983908355	3.039841397	Homo_sapiens	ENSG00000043355
WT1	1.037269516	1.697091125	2.953935065	Homo_sapiens	ENSG00000184937
FOSL1	1.032546628	1.397366589	2.800275982	Homo_sapiens	ENSG00000175592
NFE2L3	1.371167388	3.389356409	2.074785224	Homo_sapiens	ENSG00000050344
ARNTL2	1.814088635	2.244923607	1.756266603	Homo_sapiens	ENSG00000029153
TEAD4	1.904293897	1.760607232	1.686903206	Homo_sapiens	ENSG00000197905
GRHL1	1.946582032	0.92752845	1.630989319	Homo_sapiens	ENSG00000134317
MYC	1.055152424	2.186096985	1.270365792	Homo_sapiens	ENSG00000136997
DACH1	0.909830725	1.739176493	1.013779709	Homo_sapiens	ENSG00000276644
TWIST1	0.989141624	1.862761878	0.991585989	Homo_sapiens	ENSG00000122691
ZNF91	-0.852991156	-1.4933755	-1.070016266	Homo_sapiens	ENSG00000167232
ZNF704	-0.887926487	-1.868345729	-1.206257819	Homo_sapiens	ENSG00000164684
HOXA5	-1.08758917	-1.615972703	-1.502705041	Homo_sapiens	ENSG00000106004
ZFP3	-0.85646124	-1.689357175	-1.880715495	Homo_sapiens	ENSG00000180787
TFCP2L1	-0.854777456	-2.446227599	-2.043918032	Homo_sapiens	ENSG00000115112
ZBTB7B	-1.031978278	-1.117624747	-2.066603389	Homo_sapiens	ENSG00000160685
TEF	-0.866182219	-1.530461137	-2.372602512	Homo_sapiens	ENSG00000167074
MIER3	-1.284334378	-1.359472802	-2.48375564	Homo_sapiens	ENSG00000155545
ZNF575	-1.28852525	-1.624291354	-2.535810473	Homo_sapiens	ENSG00000176472
ZNF415	-1.007666704	-1.344370433	-2.622543025	Homo_sapiens	ENSG00000170954
NR5A2	-1.119867074	-3.531134691	-3.101629668	Homo_sapiens	ENSG00000116833
TCF21	-1.026551377	-1.722132158	-3.144913067	Homo_sapiens	ENSG00000118526
RFX6	-1.517582559	-1.057872221	-3.180256142	Homo_sapiens	ENSG00000185002
KLF4	-0.878855462	-2.50434832	-3.239343519	Homo_sapiens	ENSG00000136826
ZBTB7C	-1.776239225	-3.42090502	-3.397782041	Homo_sapiens	ENSG00000184828
THRB	-1.804858893	-2.589019009	-3.488621341	Homo_sapiens	ENSG00000151090
NR3C2	-1.256501239	-3.312399558	-3.546916766	Homo_sapiens	ENSG00000151623
NKX2-3	-1.044638088	-2.436903227	-3.607081348	Homo_sapiens	ENSG00000119919
ISX	-1.529523575	-2.786487964	-3.71280956	Homo_sapiens	ENSG00000175329
AFF3	-0.913297224	-1.242760499	-3.885573679	Homo_sapiens	ENSG00000144218
NEUROD1	-2.509889536	-1.171760602	-4.759083735	Homo_sapiens	ENSG00000162992
SPIB	-2.358969003	-1.975018204	-6.152089965	Homo_sapiens	ENSG00000269404

Supplementary Table S8. Immunohistochemistry analysis of ETV4 expression in colorectal cancer.

Pathological variables	Sample no.	ETV4 IHC staining(%)			P value（a）
		IRS 0	IRS 1	IRS 2	
Normal	10	0(0)	6 (60.0)	4(40.0)	0.003*
Malignant	108	3 (2.8)	15 (13.9)	90 (83.3)	
**Stage**					0.073
I＋II	69	0 (0)	9 (13.0)	60 (87.0)	
III＋IV	39	3 (7.7)	6 (15.4)	30 (76.9)	
**Lymph node status**					0.049*
pN0	71	0 (0)	9 (15.3)	62 (68)	
pN1+	37	3 (21.6)	6 (8.1)	28 (70.3)	
**Grade**					0.043*
1	37	0 (0)	6 (16.2)	31 (83.8)	
2	45	0 (0)	3 (6.7)	42 (93.3)	
3	19	1 (5.3)	5 (26.3)	13 (68.4)	
**Missing**	7				
**Tumour size**					0.706
T1					
T2	4	0 (0)	0 (0)	4(100.0)	
T3	49	1 (2.0)	9 (18.4)	39 (79.6)	
T4	53	2 (3.8)	6 (11.3)	45 (84.9)	

a. Fisher's Exact Test

*** P value<0.05**

IRS0=0； IRS1≤3； IRS2>3
